# The C‐terminal domain of yeast Arginyltransferase1 is essential for its catalytic activity

**DOI:** 10.1002/2211-5463.70336

**Published:** 2026-09-21

**Authors:** Vikas Kumar Yadav, Shweta Shweta, Manisha Malhotra, Vikash Maurya, Akhilesh Kumar

**Affiliations:** ^1^ Laboratory of Molecular Genetics and Biochemistry, Department of Botany, Institute of Science Banaras Hindu University Varanasi UP India

**Keywords:** arginylation, Arginyltransferase 1, Ate1, cell death, GNAT fold, N‐degron pathway

## Abstract

Arginyltransferase 1 (Ate1), a conserved eukaryotic enzyme, has recently been structurally characterised; identifying a conserved N‐terminal domain, a central GNAT fold and a variable C‐terminal domain of unknown function. Here, using budding yeast as an experimental model, we established that the C‐terminal domain is indispensable for Ate1 stability, its enzymatic activity and Ate1‐mediated cell death. Furthermore, we found that the co‐expression of separated N‐terminal and C‐terminal halves of Ate1 did not reconstitute enzyme activity *in vivo*. Also, we observed that the mutation of conserved residues involved in cofactor binding and active site formation within the N‐terminal half of Ate1 abolishes the enzyme function. Moreover, we showed that mouse Ate1, when heterogeneously expressed in yeast, was catalytically active but not lethal. These findings underscore the structural interdependence of Ate1 domains and their integrity in maintaining enzyme catalytic activity and stability, providing deeper insights into the enzyme structure–function relationship.

## Graphical abstract

Arginyltransferase 1 (Ate1), a eukaryotic enzyme, catalyses arginylation, transferring arginine from tRNA‐Arg to the amino terminus of the target protein. Overexpression of Ate1 in yeast is lethal and is dependent on arginylation. This study elucidates how mutations in the cofactor‐binding and active site of Ate1 and truncation of its structural domains affect the enzyme stability, activity and Ate1‐mediated cell death.

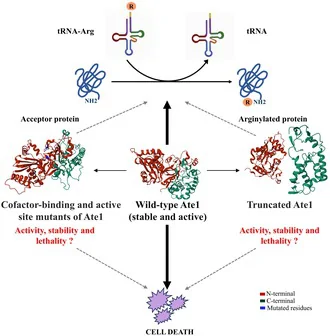

**Abbreviations**
aa: amino acid
Ate1: arginyltransferase1
GFP: green fluorescent protein
GNAT: GCN5‐related N‐acetyltransferase
PDB: protein data bank
Pgk1: phosphoglycerate kinase1
RDD: arginine‐aspartic acid‐aspartic acid
RMSD: root mean square deviation
SMART: simple modular architecture research tool

Arginyltransferase 1 (Ate1) is an aminoacyl‐tRNA transferase enzyme that is conserved among eukaryotes and regulates diverse cellular processes [1, 2, 3, 4, 5, 6, 7, 8, 9]. Ate1 catalyses the post‐translational modification known as arginylation, which involves the addition of an extra arginine molecule from charged tRNA^Arg^, preferentially to the amino terminus of a protein bearing acidic amino residues (Fig. 1A) [10, 11, 12]. The *ATE1* gene was cloned and functionally described for the first time in the budding yeast *Saccharomyces cerevisiae* and found to be nonessential, as *ate1*Δ yeast cells were viable and showed a normal phenotype [3]. However, further studies revealed the essentiality of Ate1 in higher eukaryotes, where deletion of the *ATE1* gene causes severe cardiovascular defects, including heart development and impaired angiogenesis, leading to death of mice at the embryonic stage [13].

**Fig. 1:**
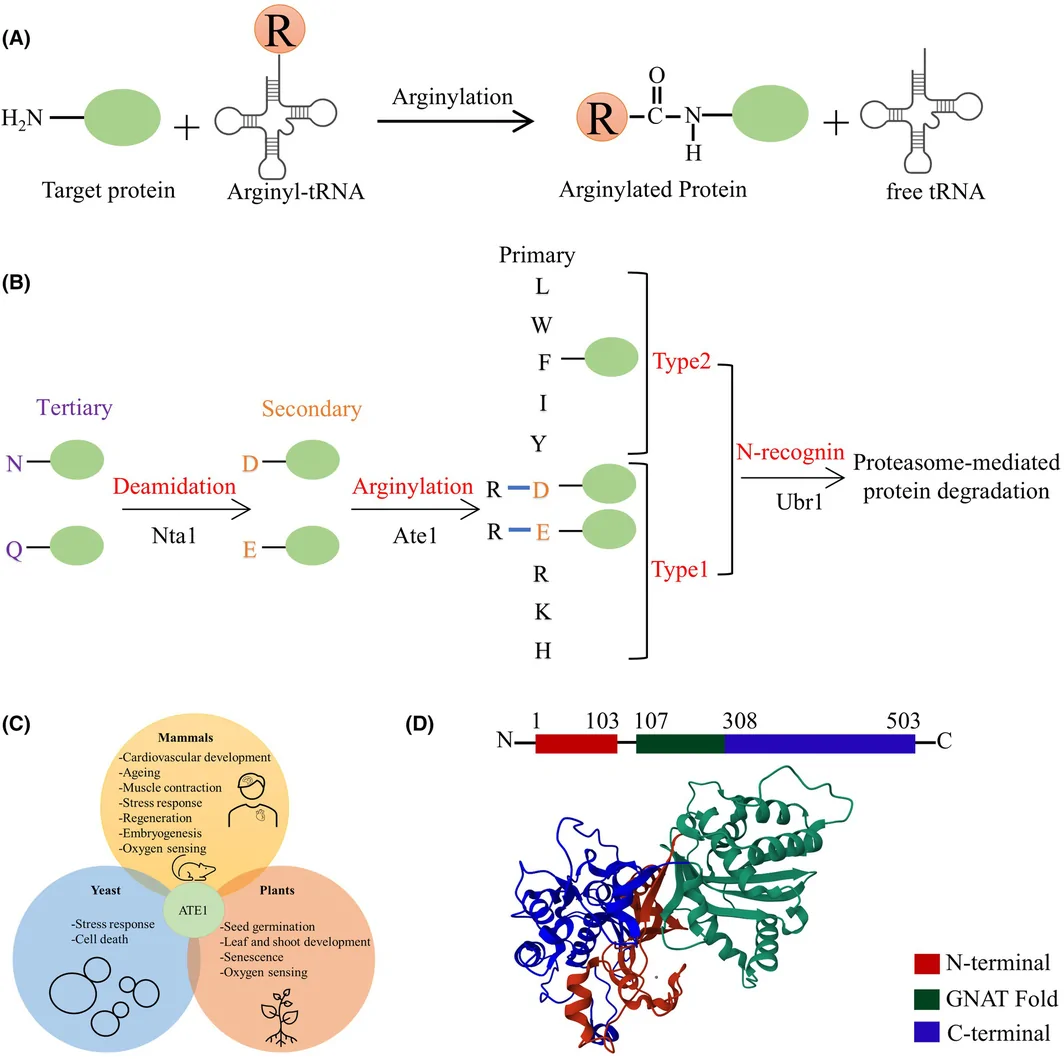
Post‐translational arginylation reaction, the Arg/N‐degron pathway, biological significance and the domain organisation of Ate1. (A) Schematic representation of the Ate1‐mediated arginylation reaction. (B) The Arg/N‐degron pathway in *Saccharomyces cerevisiae*. (C) Ate1‐catalysed post‐translational arginylation acts as a master regulator of several biological pathways across diverse eukaryotic organisms. (D) Line diagram and three‐dimensional structure of Ate1 illustrating the different functional domains of Ate1, N‐terminal domain (1‐103 aa), GNAT fold (107‐307 aa) and C‐terminal domain (308‐503 aa) highlighted with red, green and blue, respectively. Original source of the image: pdb_00008e3s, https://doi.org/10.2210/pdb8E3S/pdb.

The essentiality of Ate1‐mediated arginylation is also integral to the N‐degron pathway that regulates the turnover of proteins via the ubiquitin‐proteasome system in a cell, based on the protein's N‐terminal amino acid residue identity [12, 14, 15, 16]. Ate1 selectively recognises the proteins and peptides bearing N‐terminal tertiary (glutamine, asparagine) and secondary destabilising residues (glutamic acid, aspartic acid, or oxidised cysteine) and catalyses the transfer of arginine, a primary destabilising residue, at their N‐terminus. The resulting N‐terminal arginine acts as a strong N‐degron and is recognised by N‐recognin, especially by E3 ligases, further leading to ubiquitination and degradation of the arginylated protein via the ubiquitin‐mediated degradation pathway (Fig. 1B) [11, 17, 18, 19, 20]. By controlling the stability and fate of its target protein through the Arg/N‐degron pathway, Ate1 regulates several key physiological processes in eukaryotes, including cytoskeleton dynamics and cell motility by targeting β‐actin [4, 21, 22], G‐protein signalling involving RGS4, 5 and 7 [8, 19, 23, 24] and neurodegeneration by targeting aggregation‐prone α‐synuclein and β‐amyloid [6, 25, 26]. Besides this, Ate1 also plays a key role in regulating cancer [9, 27], ageing [5, 28], oxygen sensing [29, 30], stress response and cell death (Fig. 1C) [7, 31].

Recently, the three‐dimensional (3D) architecture of the Ate1 protein has been elucidated in yeast and human using X‐ray crystallography and cryo‐electron microscopy, respectively, which has strengthened the enzyme structure–function relationship [32, 33, 34, 35]. The structural findings show that *S. cerevisiae* Ate1 (*sc*Ate1) is a monomeric metalloprotein having a complex secondary structure made up of 22 alpha‐helices and 19 beta strands. The *sc*Ate1 comprises three major domains: N‐terminal regulatory domain (1–103aa), C‐terminal variable domain (308–503aa) and a core GNAT fold domain (107–307aa) (Fig. 1D) [35]. The N‐terminal domain of *sc*Ate1 consists of a CXXC motif, a potential Zn metal ion or Fe‐S cluster binding site, coordinated by conserved cysteine residues [30, 33]. The GNAT fold domain has a characteristic α‐β‐α sandwich where an antiparallel β‐sheet is surrounded on both faces by α‐helices. The β‐strands that make up the β‐sheet run parallel through the central protein axis and form a V‐shaped cleft, which acts as a tRNA^Arg^ binding and interaction site as well as contains the active site pocket where arginylation occurs. The active site is a deeper hydrophilic pocket that consists of conserved residues starting with a polar gate composed of Y293 and Y294 and terminating at the carboxylate side chain of D277. The polar amino acids present at the side of the active site pocket make electrostatic and hydrogen bonding interactions with D277 and Y289, while the hydrophobic residues F258 and W260 π‐stack with 3′ adenosine of amino‐acylated tRNA to carry out arginylation. The *sc*Ate1 C‐terminal domain, having an electronegative surface, is variable in nature and predicted to modulate the binding of amino‐acylated tRNA owing to the electrostatic repulsion between C‐terminal amino acid residues and RNA phosphate backbone [33, 35].

Even though the 3D structure of Ate1 has now been characterised, the prior understanding about its structure was based completely on its functional similarity with bacterial peptidoglycan synthases Fem ABX and prokaryotic leucyl phenylalanyl protein transferases (L/F transferase) [36]. These enzymes also use aminoacyl tRNA charged with a specific amino acid as a co‐substrate to catalyse the modifications of their target protein in a nonribosome‐dependent manner [37, 38, 39]. Interestingly, one more common feature is the presence of the GNAT fold within these enzymes, a conserved catalytic domain especially observed in N‐acetyl transferases (NATs) [40]. The GNAT fold is made up of a three‐layered α‐β‐α secondary structure and harbours the essential residues in the active site for the interaction of substrate with the enzyme to carry out the catalytic reaction. Similar to Ate1, the NATs also mediate N‐terminal modification of their target protein by covalently transferring the acetyl group from acetyl‐coenzyme A to the amino group of proteins and regulate their stability, interaction and turnover within the cell [41, 42]. This functional similarity, along with the GNAT fold, suggests an evolution of enzymes over time while retaining the core activity conferred by the conserved domains.

Previous studies regarding the Ate1 structure have mainly focused on the N‐terminal half comprising the N‐terminal regulatory and GNAT fold domain due to their conserved nature [32, 33]. However, the significance of the C‐terminal domain in Ate1 structure and function is not yet clear due to the lack of sequence homology in this domain. Thus, we investigated the structural and functional importance of this domain.

For this purpose, we initially performed bioinformatics analysis of *sc*Ate1, which revealed that the N‐terminal half is highly conserved and consists of a CXXC motif and active site residues involved in Ate1 substrate recognition and tRNA^Arg^ interaction. However, it did not disclose any apparent function of the C‐terminal domain due to its less conserved nature. Next, using mutation and truncation experiments, we elucidated that these conserved residues present in the N‐terminal region as well as the C‐terminal domain of Ate1 are important for stability, arginylation and Ate1‐mediated cell death in yeast. Further, we have also shown that not only stability but possibly cotranslational protein folding is a prerequisite for Ate1's catalytic activity, as the *in vivo* co‐expression of separated N‐terminal and C‐terminal halves of Ate1 does not reconstitute enzyme activity. Further, we have observed that the C‐terminal domain has significantly evolved in mouse Ate1, which is catalytically active but nonlethal in yeast, suggesting the different substrate specificity and subcellular localisation of mouse and yeast Ate1 within the cell. Overall, our findings conclude that the variable C‐terminal domain, as well as its structural integrity to the N‐terminal domain and GNAT fold, is essential for Ate1 stability, activity and Ate1‐mediated cell death in yeast.

## Materials and methods

### Growth and maintenance of *E. coli* and yeast strains

The bacterial strain *E. coli* DH5α was chosen as a suitable host for our molecular cloning purpose. Luria–Bertani (LB) broth, consisting of 5 g·L^−1^ yeast extract, 10 g·L^−1^ peptone, 10 g·L^−1^ NaCl and 1% agar (optional), was used as a growth medium for maintenance of bacterial cells at 37 °C [43]. Yeast strains and plasmids used in this study are mentioned in Tables S1 and S2, respectively. YPD, a nutrient‐rich media containing 10 g·L^−1^ yeast extract, 20 g·L^−1^ peptone, 20 g·L^−1^ dextrose and 2% agar (optional), was used to grow the yeast cells, and the culture was maintained at 30 °C [44].

### Molecular cloning and site‐directed mutagenesis

All molecular cloning and site‐directed mutagenesis experiments were performed following standard molecular biology protocols using high‐fidelity Pfu DNA polymerase (Cat# F530S; Thermo Fisher Scientific, Mumbai, Maharashtra, India). The gene of interest was amplified by PCR using suitable primers (listed in the Table S3) obtained from Eurofins (Doddanakundi, Bengaluru, India) and appropriate templates. The PCR product and the corresponding vector were digested with compatible restriction enzymes (Thermo Fisher Scientific) and subsequently ligated into the vector using T4 DNA ligase (Cat# EL0011; Thermo Fisher Scientific). The ligation mixture was transformed into *Escherichia coli* competent cells, and positive clones were selected on Luria–Bertani agar plates supplemented with ampicillin. PCR reactions were performed in a T100 thermal cycler (Bio‐Rad, Gurugram, Haryana, India). Details of all cloned genes and site‐directed mutagenesis constructs (verified by Sanger sequencing—refer to Data S1) are provided below:

- Ate1‐C20S

To mutate the conserved cofactor‐binding site residue cysteine (C20) into serine (S20) in the CXXC motifs of the Ate1, site‐directed mutagenesis was carried out on the pYES‐ATE1‐GFP plasmid, which served as a template, using two sets of primers. PCR1 was conducted using Ate1‐HindIII‐F and Ate1‐C20S‐R primers, while PCR2 was carried out using Ate1‐C20S‐F and Ate1‐EcoRI‐R primers. PCR1 and PCR2 products were combined in equimolar amounts and used as a template for creating the C20S mutation. A third PCR, using Ate1‐HindIII‐F and Ate1‐EcoRI‐R primers, amplified the *ATE1* with the C20S mutation. The mutated *ATE1* with the C20S mutation was cloned between HindIII and EcoRI restriction sites present within the pYES‐ATE1‐GFP plasmid.

- Ate1‐C23S

To mutate the conserved cofactor‐binding site residue cysteine (C23) into serine (S23) in the CXXC motifs of the Ate1, site‐directed mutagenesis was carried out on the pYES‐ATE1‐GFP plasmid, which served as a template, using two sets of primers. PCR1 was conducted using Ate1‐HindIII‐F and Ate1‐C23S‐R primers, while PCR2 was carried out using Ate1‐C23S‐F and Ate1‐EcoRI‐R primers. PCR1 and PCR2 products were combined in equimolar amounts and used as a template for creating the C23S mutation. A third PCR, using Ate1‐HindIII‐F and Ate1‐EcoRI‐R primers, amplified the *ATE1* with the C23S mutation. The mutated *ATE1* with the C23S mutation was cloned between HindIII and EcoRI restriction sites present within the pYES‐ATE1‐GFP plasmid.

- Ate1‐C20,23S

To mutate the conserved cofactor‐binding site residue cysteine (C20, C23) into serine (S20, S23) in the CXXC motifs of the Ate1, site‐directed mutagenesis was carried out on the pYES‐ATE1‐GFP plasmid, which served as a template, using two sets of primers. PCR1 was conducted using Ate1‐HindIII‐F and Ate1‐C20S‐C23S‐R primers, while PCR2 was carried out using Ate1‐C20S‐C23S‐F and Ate1‐EcoRI‐R primers. PCR1 and PCR2 products were combined in equimolar amounts and used as a template for creating the C20‐C23 mutation. A third PCR, using Ate1‐HindIII‐F and Ate1‐EcoRI‐R primers, amplified the *ATE1* with the C20‐C23 mutation. The mutated *ATE1* with the C20‐C23 mutation was cloned between HindIII and EcoRI restriction sites present within the pYES‐ATE1‐GFP plasmid.

- Ate1‐D277A

To mutate the conserved active site residue aspartic acid (D277) to alanine (A277) in the Ate1, site‐directed mutagenesis was performed using plasmid pYES‐ATE1‐GFP as a template and two sets of primers. First, PCR1 was performed using the primers Ate1‐HindIII‐F and reverse Primer D277A. Concurrently, PCR2 was performed using Forward Primer D277A and Ate1‐SacI‐R. The resulting PCR products, PCR1 and PCR2, were combined in equimolar concentrations to serve as the template for creating the D277A mutation. Finally, a third PCR, using Ate1‐HindIII‐F and Ate1‐SacI‐R primers, was set up to amplify the *ATE1* bearing the D277A mutation successfully. *ATE1* with the mutated D277A was cloned between HindIII and SacI restriction sites present within the pYES‐ATE1‐GFP plasmid.

- Ate1‐D277K

To mutate the conserved active site residue aspartic acid (D277) to the basic residue lysine (K277) in the Ate1, site‐directed mutagenesis was performed using plasmid pYES‐ATE1‐GFP as a template and two sets of primers. First, PCR1 was performed using the primers Ate1‐HindIII‐F and reverse primer D277K. Concurrently, PCR2 was performed using forward primer D277K and Ate1‐SacI‐R. The resulting PCR products, PCR1 and PCR2, were combined in equimolar concentrations to serve as the template for creating the D277K mutation. Finally, a third PCR, using Ate1‐HindIII‐F and Ate1‐SacI‐R primers, was set up to amplify the *ATE1* bearing the D277K mutation successfully. *ATE1* with the mutated D277K was cloned between HindIII and SacI restriction sites present within the pYES‐ATE1‐GFP plasmid.

- Cloning of GFP‐tagged truncated Ate1 (1‐313aa)

From the plasmid pYES‐ATE1‐GFP, the 1–313aa region of Ate1 was amplified using forward and reverse primers Trunc. Ate1‐EcoRI‐F and Trunc.Ate1‐313aa‐XhoI‐R respectively. The amplicon was then cloned between the EcoRI and XhoI restriction sites under the GAL1 promoter in the pYES‐ATE1‐GFP vector backbone.

- Cloning of GFP‐tagged truncated Ate1 (1‐364aa)

From the plasmid pYES‐ATE1‐GFP, the 1–364 aa region of Ate1 was amplified using forward and reverse primers Trunc. Ate1‐EcoRI‐F and Trunc. Ate1‐364aa‐Xho1‐R respectively. The amplicon was then cloned between the EcoRI and XhoI restriction sites under the GAL1 promoter in the pYES‐ATE1‐GFP vector backbone.

- Cloning of GFP‐tagged truncated Ate1 (314‐503aa)

From the plasmid pYES‐ATE1‐GFP, the 314–503aa region of Ate1 was amplified using forward and reverse primers Trunc. Ate1 314_503aa‐HindIII‐F and Trunc. Ate1 314_503aa‐Xho1‐R, respectively. The amplicon was then cloned between the HindIII and XhoI restriction sites under the GAL1 promoter in the pYES‐ATE1‐GFP vector backbone.

- Cloning of GFP‐tagged truncated Ate1 (365‐503aa)

From the plasmid pYES‐ATE1‐GFP, the 365‐503aa region of Ate1 was amplified using forward and reverse primers ATE1‐365aa‐EcoRI‐F and ATEGFP‐SalI‐R, respectively. The amplicon was then cloned between EcoRI and SalI restriction sites under the *GAL1* promoter in the pRS423 vector backbone.

- Cloning of N and C‐terminal domains of Ate1

The N‐terminal domain of Ate1 (amino acids 1‐313) was amplified from the source plasmid pYES‐ATE1‐GFP and cloned under the *GAL1* promoter at the SpeI and SmaI restriction sites in the pRS423 vector carrying a His selection marker using the primers Trunc. Ate1‐313aa‐SpeI‐F and Trunc. Ate1‐313aa‐SmaI‐R. A stop codon (TGA) was introduced immediately downstream of the 313th amino acid to terminate protein translation. Similarly, the C‐terminal domain of Ate1 (amino acids 314‐503) was amplified from the same source plasmid pYES‐ATE1‐GFP and cloned at the SpeI and SmaI restriction sites in a plasmid containing the *GAL1* promoter and Ura selection marker. A start codon (ATG) was introduced immediately upstream of the 314th amino acid to initiate protein translation.

- Cloning of yeast two‐hybrid plasmids

Y2H expression plasmids were constructed by fusing the N‐terminal and C‐terminal Ate1 with GAL4 activation domain and GAL4 DNA‐binding domain, respectively. Using the plasmid pYES‐ATE1‐GFP as a template, the N‐terminal region 1‐313aa of Ate1 was amplified using forward and reverse primers Trunc. ATE1‐1‐313aa‐SmaI‐F and Trunc.Ate1‐313aa‐XhoI‐R, respectively. The resulting amplicon was then cloned into the pGADT7 vector between the SmaI and XhoI restriction sites in frame with GAL4 AD. The C‐terminal region 314–503aa was PCR amplified using the same source plasmid pYES‐ATE1‐GFP as a template and the primers Trunc. Ate1–314 to 503aa‐EcoRI‐F and Trunc. Ate1 503aa‐SalI‐R. The amplified fragment was cloned between EcoRI and SalI restriction sites in the pGBKT7 plasmid in frame with GAL4 BD.

- Cloning of mouse Ate1

To clone mouse Ate1 (mAte1), the gene was amplified from the previously described plasmid construct, containing the coding sequence of mouse Ate1 isoform 1 [7], using the mAte1.1‐BamHI‐F and mAte1.1‐XhoI‐R primers. The amplified product was then cloned between BamHI and XhoI restriction sites under the control of the GAL1 promoter into the yeast expression vector pYES2.

### *E. coli* and yeast transformation

Bacterial transformation was done using the heat shock method [45]. The chemically competent cells prepared by the CaCl_2_ method were thawed on ice for 10–15 min. Then an appropriate amount of plasmid or ligation mixture was added and mixed thoroughly and then kept on ice for 30 min. After incubation, the cells were subjected to heat shock by placing them in a water bath set at 42 °C for 90 s and quickly plunged into ice for 30 s. Subsequently, for recovery and the growth of the cells, 900 μL of LB broth was added, and the tube was placed in an incubator shaker at 37 °C, 200 rpm for 90 min. Then, the cells were plated on an LB agar plate containing an appropriate antibiotic (ampicillin, 100 μg·mL^−1^) and incubated at 37 °C overnight to allow the growth of recombinants.

Yeast transformation was carried out using the lithium acetate (LiAc) method [46]. In brief, yeast cells were treated with lithium acetate to induce DNA uptake, followed by the addition of salmon sperm single‐stranded carrier DNA and the plasmid or DNA fragment. The cell mixture was subsequently treated with polyethylene glycol (PEG) to facilitate DNA uptake into yeast cells. After the cells were heat‐shocked to increase transformation efficiency, they were plated on selection plates, SD medium, comprised of 1.7 g·L^−1^ yeast nitrogen base (YNB), 5 g·L^−1^ ammonium sulphate, 20 g·L^−1^ dextrose and 80 mg·L^−1^ essential amino acids (e.g. methionine, histidine, leucine and uracil as required) and incubated at 30 °C for growth [44].

### Bioinformatics analysis

To perform multiple sequence alignment (MSA) using clustal omega (https://www.ebi.ac.uk/jdispatcher/msa/clustalo, RRID:SCR_001591), the protein sequences of Ate1 of different groups of organisms were retrieved from the UniProt protein database (https://www.uniprot.org/, RRID:SCR_002380) and pasted into clustal omega to obtain the alignment. The MSA data were visualised and edited using bioedit. Simple Modular Architecture Research Tool (SMART), a web resource (http://smart.embl.dea), was used to identify the conserved domains within the protein. The sequence and structural information were retrieved from GenBank and Protein Data Bank (PDB), respectively. The deposited three‐dimensional structures of Ate1, having PDB: 8E3S, 8FZR, 8TZV and AlphaFold ID: AF‐Q9Z2A5‐F1‐v6, were annotated using the RCSB Protein Data Bank (PDB). Then, the pairwise structural alignment was done using the jFATCAT alignment method in RCSB PDB (https://www.rcsb.org/). The ExPASy PeptideCutter tool was used for the identification of potential protease cleavage sites in the Ate1 protein sequence.

### Serial dilution and spotting assay

Yeast transformants carrying the desired plasmid were inoculated into a selective medium, and the culture was grown overnight in an incubator shaker at 180 rpm at 30 °C. The following day, the cells were pelleted down by centrifugation at 4222 ***g***. The retained pellet was washed 2–3 times with sterile water. After washing, the cells were resuspended in sterile water, and the optical density was measured. The serial dilutions of the following optical densities (OD; 600 nm) 0.2, 0.02, 0.002 and 0.0002 were prepared, and the aliquots of 10 microliters from each dilution were spotted on selective agar plates. The plates were kept upright for 30 min so that yeast cells could adhere to the agar. After drying, the plates were maintained at 30 °C for 3–4 consecutive days to observe the growth pattern of yeast cells.

### *In vitro* arginylation assay

To perform the *in vitro* arginylation assay, we used the substrate protein DD‐β15‐GFP, a well‐known Ate1 substrate consisting of 15 amino acids derived from the mammalian actin sequence. The sequence is linearly fused with ubiquitin at the amino terminus and tagged with GFP at the carboxyl terminus. The presence of aspartic acid at its N terminus made it susceptible to arginylation by the Ate1 enzyme (Fig. S3) [47].

The plasmids cloned with the substrate protein DD‐β15‐GFP (pGPD2‐β‐reporter) and with Ate1 (pYES‐ATE1‐GFP) and its variants (mutated variant—C20S, C23S, C20,23S, D277A, D277K; truncates carrying amino acids Ate1‐1‐313, 314–503, 1–364, 365–503) were separately transformed and expressed into the *ate1*Δ yeast strain to carry out an *in vitro* arginylation assay.

Yeast transformants were grown in the selective medium containing glucose as a carbon source until the cells reached an OD of 0.6. The *ate1*Δ cells (A_600_ = 0.6) transformed with a plasmid having *a GAL1* promoter were further transferred into galactose medium for 18 h to induce the expression of the downstream cloned gene. The yeast cells were harvested and resuspended in the arginylation buffer (25 mm KCl, 50 mm HEPES (pH 7.5), 2.5 mm ATP, 0.2 mm arginine, 15 mm MgCl_2_ and 5 mm PMSF). To obtain the cell lysate, the yeast cells were disrupted using glass beads, followed by vortexing and retention of the supernatant after centrifugation at 13 000 rpm at 4 °C. The cell lysate from *ate1*Δ cells expressing the substrate protein and Ate1 were mixed in equal volume, and the mixture was then incubated at 30 °C for 1 h to initiate the arginylation reaction. Further, 1/4 volume of 5X SDS sample buffer was added to the mixture to stop the reaction, following the incubation in a water bath at 100 °C for 10 min. After incubation, the centrifugation was done at 15 873 ***g*** for 5 min to pellet any debris. Next, a 12% SDS‐polyacrylamide gel was run, and immunoblotting was performed to detect the arginylation signal [7].

### SDS/PAGE and western blotting

To run the SDS/PAGE, 12% resolving gel and 5% stacking gel were prepared. The protein samples were loaded in their respective wells along with the protein ladder, and the gel was run to separate the proteins based on their molecular weight. Following separation, the proteins were transferred from the gel to the nitrocellulose membrane. After transfer, Ponceau staining was done to ensure proper transfer, followed by blocking, washing and incubating them with the primary and secondary antibodies, respectively. 5% skimmed milk (Cat# GRM1254; Himedia Laboratories, Thane, Maharashtra, India) was used as a blocking reagent to minimise the background noise and nonspecific binding of antibodies. The primary antibodies used in this study were as follows: a polyclonal rabbit anti‐GFP antibody (Cat# A01388‐40; G2P, Secunderabad, Hyderabad, India), to detect GFP; a monoclonal rat anti‐Ate1 antibody (Cat# MABS436) to detect the mouse Ate1; and a monoclonal anti‐mouse Pgk1 antibody (Cat# 459250, RRID:AB_2532235; Invitrogen, Bangalore, Karnataka, India), to detect Pgk1. HRP‐conjugated secondary antibodies specific to rabbit, rat and mouse were used for the detection of the primary antibodies. After probing, the protein bands specific to our protein were visualised by developing the blot using the chemiluminescence kit (Cat# 1705061; ECL Bio‐Rad) as per the manufacturer's instructions, and the produced signals were detected with the bio‐rad molecular imager ChemiDoc XRS+ imaging system.

### Yeast‐2 hybrid assay

To examine the interaction between the N‐ and C‐terminal regions of Ate1, expression plasmids were constructed by fusing the N‐terminal and C‐terminal Ate1 with the GAL4 activation domain in pGBKT7, BD vector and the GAL4 DNA‐binding domain in pGADT7, AD vector, respectively. Using the lithium acetate transformation method, these constructs were cotransformed in the AH109 strain in different combinations along with positive and negative controls. The yeast transformants were selected on SD medium lacking leucine and tryptophan to confirm the presence of both vectors. To determine the protein interaction, a spotting assay was performed using 2D and 4D (‐leu‐trp‐ade‐his) selection plates. The plates were incubated at 30 °C for 3 days.

## Results

### Ate1 protein sequence and structure analysis do not reveal any obvious function of its C‐terminal variable domain

**Fig. 2:**
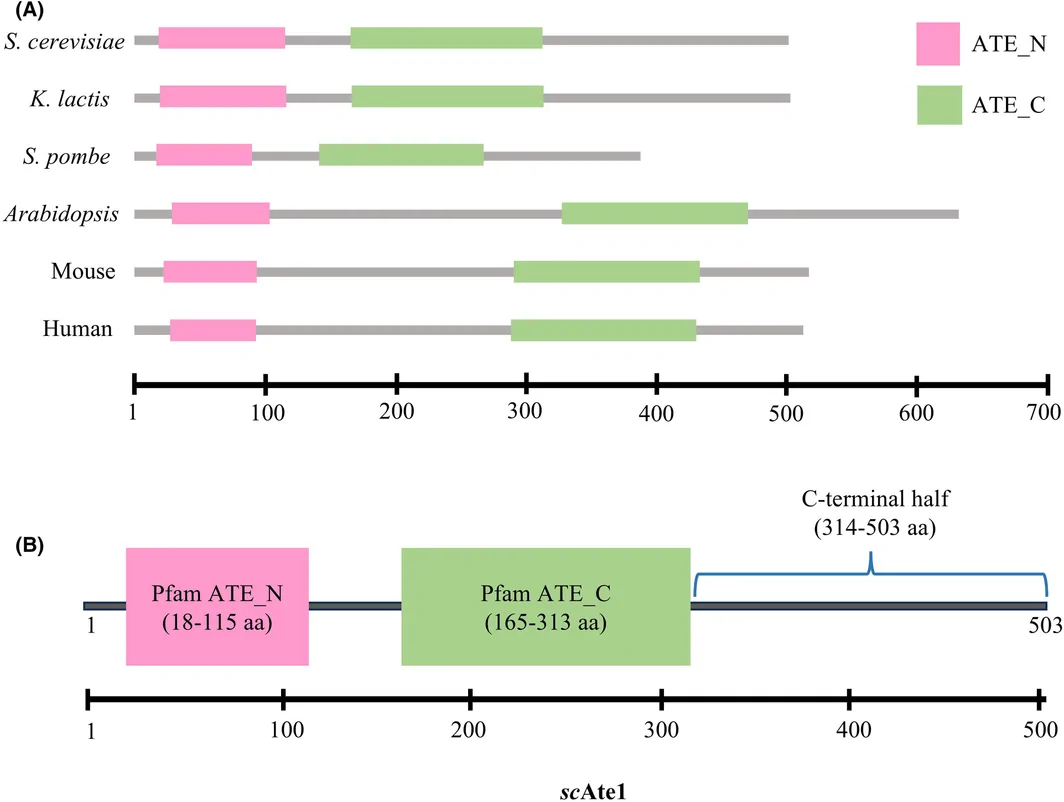
Conserved Pfam domain analysis of Ate1. (A) Pfam domain analysis using SMART reveals the presence of two conserved domains in Arginyltransferase1, namely Ate1_N and Ate1_C, across different eukaryotes. (B) Pfam domain organisation of the *sc*Ate1 protein; each rectangle represents a distinct Pfam domain. The colours assigned are as follows: pink and green represent the ATE_N and ATE_C domains, respectively. The amino acid sequence is numbered to highlight where each domain begins and ends.

The conservation of specific amino acid residues or protein domains across different species often indicates their functional and evolutionary significance. In our study, we conducted a comprehensive bioinformatics analysis at the domain, structural and sequence level to elucidate the importance of the C‐terminal domain of Ate1. Firstly, we conducted Pfam domain analysis using SMART, where we identified two conserved Pfam domains in the Ate1 enzyme, namely ATE_N and ATE_C (Fig. 2A and Fig. S1). In the case of budding yeast *S. cerevisiae* Ate1 (*sc*Ate1), the conserved Pfam domains were present in the N‐terminal half (comprising the N‐terminal and GNAT fold domains), spanning amino acids 18‐115 and 165‐313, respectively, implying a potentially high degree of conservation. Contrary to this, the C‐terminal half (further annotated as C‐terminal variable domain) of *sc*Ate1 encompasses residues 314 to 503 and lacks any Pfam annotation, suggesting that this region is evolutionarily less conserved than other regions of the protein (Fig. 2B).

**Fig. 3:**
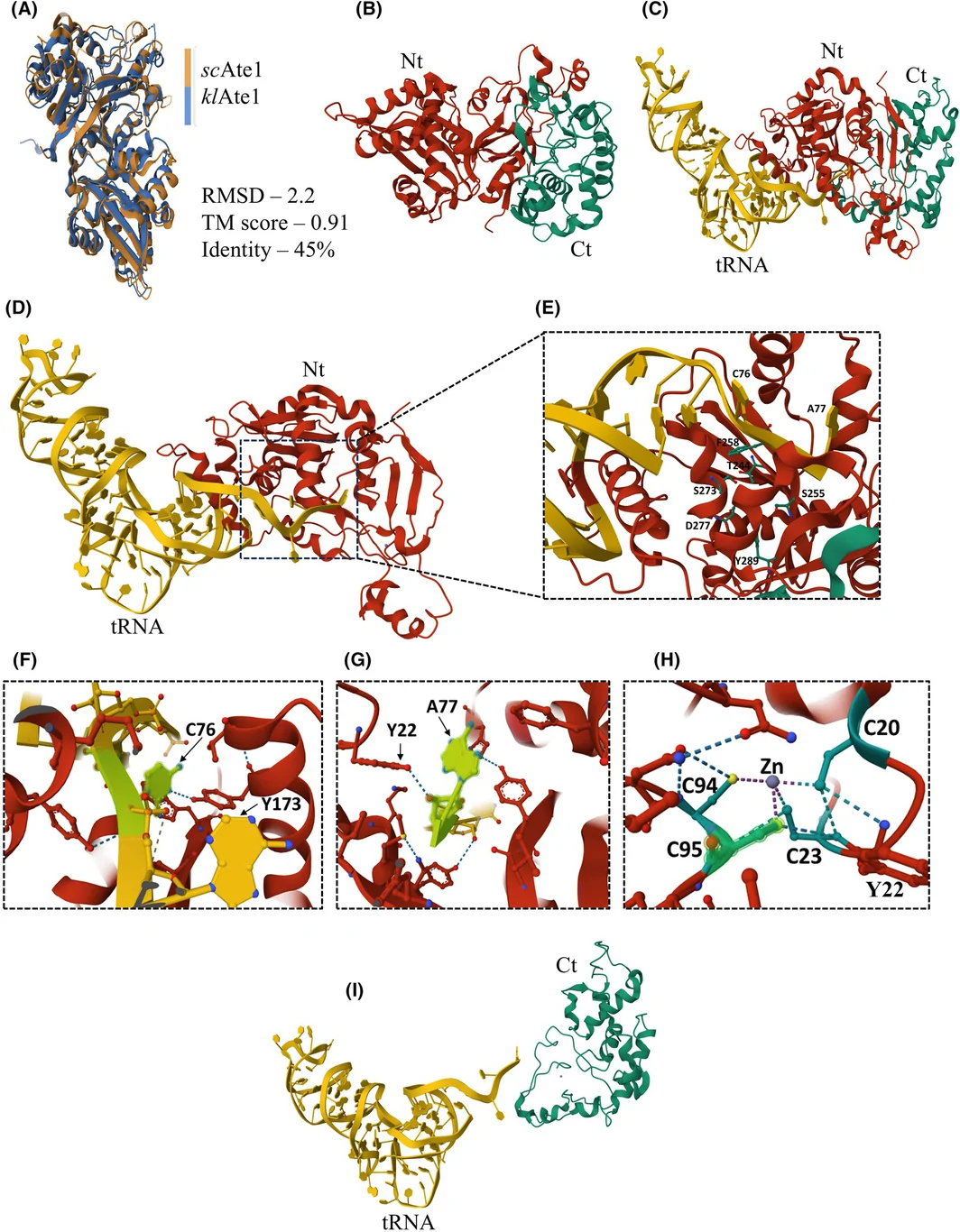
Structural analysis of Ate1. (A) Superimposed three‐dimensional structure of *sc*Ate1 (PDB: 8E3S) with *k*lAte1 (PDB: 7WG4). Cartoon representation of *sc*Ate1 in (B) apo form (PDB: 8E3S) and (C) tRNA^Arg^ bound form (PDB: 8FZR) with N‐terminal half, C‐terminal half and tRNA highlighted in red, green and yellow, respectively. (D) 3D structure of *sc*Ate1 with hidden C terminus, (E) enlarged view of *sc*Ate1 N‐terminal half showing the active site pocket and its residues, which comprise the inferred site that interacts with tRNA^Arg^ cofactor. (F, G) An enlarged view illustrating the hydrogen bonding (blue colour dotted line) between the amino acid residues Y22 and Y173 in the N‐terminal half of *sc*Ate1 with two terminal nucleotides A77 and C76 of tRNA^Arg^. (H) Close‐up of the N‐terminal half revealing an atypical zinc‐finger motif coordinated by amino acid residues C20, C23, C94 and C95 to form a conserved cysteine cluster. The purple colour dotted lines represent the metal coordinate bonds. (I) 3D structure of Ate1 with a hidden N terminus, showing that the C‐terminal half does not directly interact with tRNA^Arg^. Original source of the image: pdb_00008e3s, https://doi.org/10.2210/pdb8E3S/pdb; pdb_00007wg4, https://doi.org/10.2210/pdb7WG4/pdb; pdb_00008fzr, https://doi.org/10.2210/pdb8FZR/pdb.

As the structure of the Ate1 enzyme has already been determined in yeast *S. cerevisiae* and *Kluyveromyces lactis*, we performed rigid‐body protein structure alignment to reveal the potential conserved structural features among them. The superimposed structure obtained has a root mean square deviation (RMSD) value of 2.2 angstroms, indicating a good fit between the two protein structures. This analysis was further supported by a template modelling (TM) score of 0.91, which was close to 1 and is indicative of a near‐perfect structural match (Fig. 3A). Next, to gain a more detailed view of the structural variations present between different domains of *sc*Ate1, we analysed its apo and tRNA‐bound form in depth (Fig. 3B,C).

We found that the GNAT fold, tRNA^Arg^ binding site and zinc‐finger motif essential for catalytic activity of *sc*Ate1 were present in the N‐terminal half of Ate1, and the variable C‐terminal domain appears functionally ambiguous in these interactions. The GNAT fold consists of an active site pocket bearing hydrophobic residues Y289, S255, T244, S273, F258 and negatively charged D277, which interacts with the major groove of the acceptor arm of the tRNA^Arg^ (Fig. 3D,E). The nucleotides C76 and A77 present at 3′end of tRNA^Arg^ form hydrogen bonds with the tyrosine residue present at position Y173 and Y22, respectively (Fig. 3F,G) and are proximal to the conserved CXXC zinc‐finger motif, which contributes to the substrate binding domain (Fig. 3H). Overall analysis indicates that the N‐terminal half is highly conserved and essential for the catalytic activity of *sc*Ate1, while low sequence homology and lack of interaction of the C‐terminal domain with tRNA^Arg^ suggest no obvious role of this domain in enzyme activity (Fig. 3I).

Furthermore, we retrieved the Ate1 protein sequence of various organisms and performed multiple sequence alignment (MSA) using Clustal Omega. We observed that *sc*Ate1 shares 45, 30, 28, 31 and 29% sequence similarity with *K. lactis*, *Schizosaccharomyces pombe*, *Arabidopsis thaliana*, mouse, and human, respectively. The MSA identified blocks of conserved residues in the N‐terminal half of Ate1, while the C‐terminal half shows significant variability, pointing out that the N‐terminal half of Ate1 is conserved during evolution in eukaryotes and may be responsible for the protein function and structure (Fig. S2). The bioinformatics analysis corroborates past studies that the N‐terminal and GNAT fold domain is highly conserved and harbours an active site and cofactor‐binding site as well as interacts with tRNA^Arg^, while there is no apparent role of the C‐terminal domain of *sc*Ate1 due to its variable sequence.

### Mutations of the conserved cofactor‐binding cysteine cluster and active site residues of Ate1 lead to loss of its arginylation activity and toxicity

The structural data of the Ate1 enzyme revealed that the conserved CXXC zinc‐finger motif, coordinated by four cysteine residues (C20, C23, C94 and C95), lies within the N‐terminal domain. To examine how the mutation of conserved cofactor‐binding cysteine residues affects Ate1 catalytic activity and cell death, we selected two cysteine residues, C20 and C23, and performed site‐directed mutagenesis to change either C20 or C23 or both into serine in *Ate1‐GFP* carried by a plasmid pYES‐Ate1‐GFP having *GAL1* promoter. Next, we transformed the GFP‐tagged Ate1 mutant constructs into the *ate*1∆ yeast to perform the *in vitro* arginylation assay.

To examine the effect of these mutations on the arginylation activity of Ate1, we used a linear ubiquitin‐tagged arginylation reporter protein (DD‐β15‐GFP) based on the ubiquitin‐fusion technique and performed the *in vitro* arginylation assay. The arginylation reporter consists of a 15‐amino acid beta‐actin sequence flanked by ubiquitin at the amino terminus and GFP at the carboxyl terminus. The expression of this reporter within eukaryotes leads to co‐translational deubiquitylation of the reporter protein by the endogenous deubiquitylation enzymes, resulting in exposure of the aspartic amino acid at the N terminus. The exposed aspartic amino acid, being a destabilising residue, undergoes arginylation by the Ate1 enzyme (Fig. S3) [7, 47].

For our study, we performed *in vitro* arginylation by incubating the *ate1∆* cell lysate expressing the arginylation reporter with the *ate1∆* cell lysate expressing either the wild‐type Ate1 or its specific mutants (Ate1‐C20S, Ate1‐C23S, Ate1‐C20,23S). After arginylation, immunoblotting was done, and the membrane was probed with anti‐RDD antibody to detect the arginylation signal and with anti‐GFP antibody to detect the stability of GFP‐tagged Ate1 constructs and to assess the steady‐state level of the reporter substrate (β‐rep‐GFP) present within the cell.

**Fig. 4:**
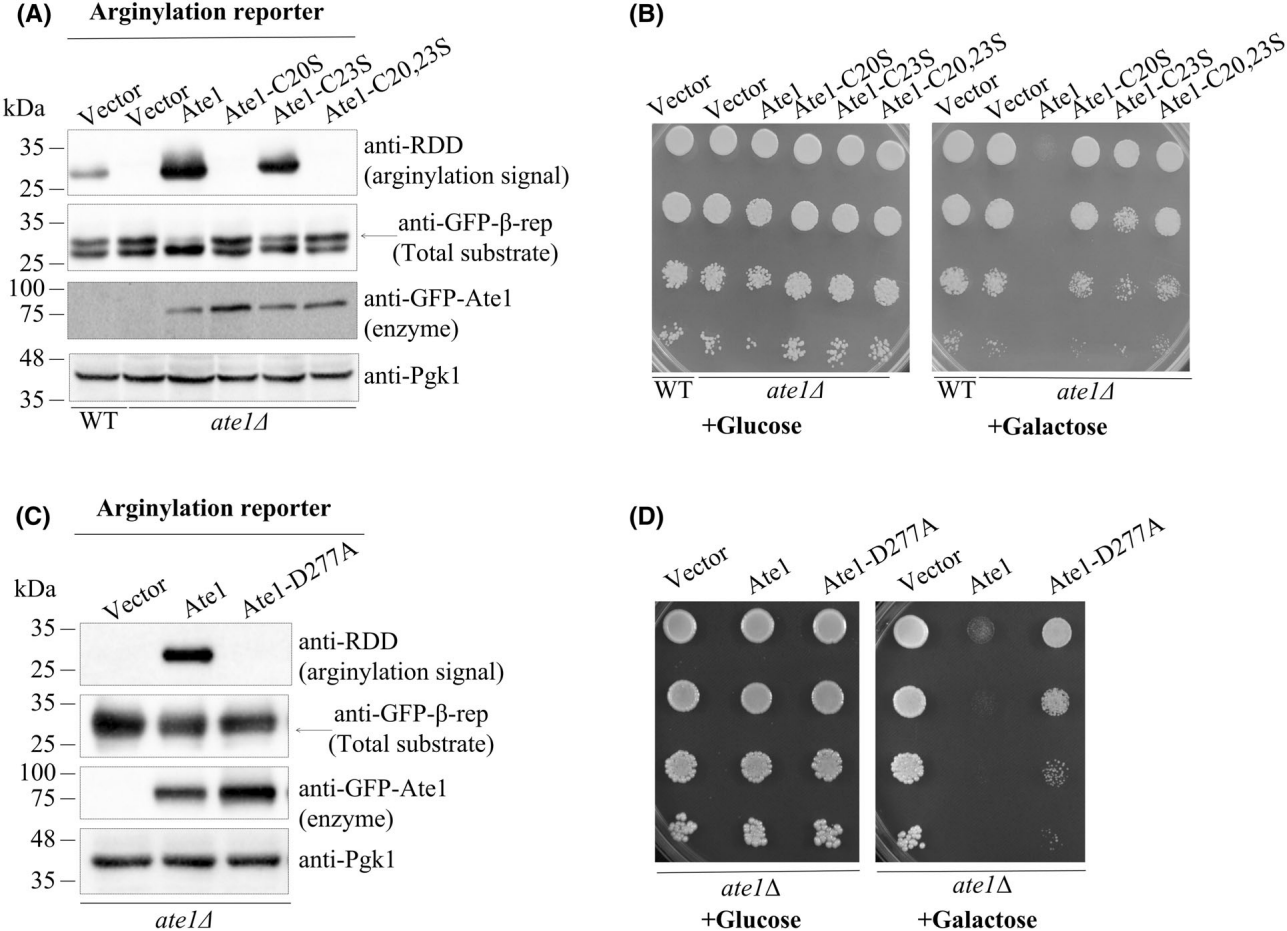
Mutation of conserved amino acid residues lying in the N‐terminal half of Ate1 abolished arginylation activity and Ate1‐mediated cell toxicity. (A, C) Immunoblots showing arginylation and steady‐state levels of the Ate1 substrate—DD‐β15‐GFP (arginylation reporter) in *ate1Δ* cells expressing the wild‐type or indicated mutants of Ate1. The stability of GFP‐tagged Ate1 constructs was assessed using an anti‐GFP antibody. The *ate1*Δ yeast cell transformed with the vector acts as a negative control, while wild‐type cells transformed with the vector act as a positive control for this *in vitro* arginylation assay. Pgk1, a cytosolic enzyme, was used as a loading control. (B, D) Spotting assay depicting the growth pattern of *ate1*Δ yeast cells transformed with the vector, wild‐type Ate1 and the mentioned Ate1 mutants on SD‐glucose (left panel, repressible condition) and SD‐galactose (right panel, inducible condition) plates, respectively.

Immunoblot data reveal that the anti‐RDD signal, which represents the level of arginylation, was present in *ate1*∆ cells expressing wild‐type Ate1 and Ate1‐C23S; however, the arginylation band intensity was weaker in Ate1‐C23S as compared to the wild‐type. Interestingly, the anti‐RDD signal was completely absent in *ate1*∆ cells expressing Ate1‐C20S and Ate1‐C20,23S. Furthermore, we observed that the β‐rep‐GFP band intensity was higher in nonarginylated substrates than in arginylated substrates in *ate1*∆ cells, confirming that the lack of arginylation leads to increased stability of the arginylation reporter. Here, Pgk1 was used as a loading control, and the wild‐type and *ate1*∆ cells transformed with the vector served as positive and negative controls for this *in vitro* arginylation assay. Next, we checked the expression levels of wild‐type and mutant Ate1 constructs tagged with GFP and found that their expression was similar, which suggests that the partial and complete loss of arginylation signal in *ate1*∆ cells expressing Ate1‐C23S, Ate1‐C20S and Ate1‐C20,23S mainly arises from impaired enzymatic activity postmutation, rather than altered expression of the Ate1 protein. Also, C20 was found to be more important than C23 for arginylation, as the C20S mutation leads to loss of Ate1 activity, while in the C23S mutation, Ate1 was able to partially retain its enzymatic activity (Fig. 4A, Table S4). Previous studies have shown that the ectopic expression of Ate1 leads to cell death in yeast [7]. Thus, to further investigate the importance of the CXXC motif in Ate1‐mediated cell death, a spotting assay was done. We observed that the overexpression of wild‐type Ate1 was lethal as expected, while Ate1‐C23S was less toxic compared to the wild‐type. However, Ate1‐C20S and Ate1‐C20,23S were found to be nonlethal and completely rescued the *ate1*∆ cells from Ate1‐mediated toxicity (Fig. 4B). Together, the immunoblot and cell death assay suggest that the C20 amino acid residue is more important than C23 in the CXXC motif for Ate1 enzymatic activity and Ate1‐mediated cell death, as mutation at C20S either alone or in combination with C23S completely abolishes the Ate1 arginylation activity and the toxic effects associated with Ate1 overexpression.

Verna Van has proposed an arginylation mechanism in 2022, where they reported that D277, Y293, and R79/80 amino acid residues are likely to be involved in the Ate1‐mediated post‐translational arginylation reaction [35]. In the *sc*Ate1.tRNA^Arg^ structure as well, D277 was shown to be located in proximity to the enzyme's active site pocket interacting with tRNA. Thus, we mutated the amino acid D277 into a nonpolar amino acid, alanine, in *Ate1‐GFP* carried by a plasmid pYES and expressed the wild‐type and mutated Ate1 constructs in *ate1*∆ cells. We performed *in vitro* arginylation followed by immunoblotting as mentioned earlier and found that the anti‐RDD arginylation signal was present in *ate1∆* cells carrying wild‐type Ate1 and was completely absent in *ate1∆* cells carrying the Ate1‐D277A mutation. To check whether this mutation affects arginylation due to structural disruption or due to a change in its side chain, we examined the expression of GFP‐tagged wild‐type and mutant Ate1. We observed that the expression of Ate1‐D277A was similar to the wild‐type, suggesting that the mutation of aspartic acid into alanine abolished enzymatic activity rather than protein stability (Fig. 4C, Table S4). Next, to examine how the expression of mutant Ate1‐D277A would affect yeast growth, a serial dilution‐based cell death assay was performed. The assay shows that *ate1∆* cells carrying either vector or wild‐type Ate1 or mutant Ate1‐D277A show a similar growth pattern under repressible conditions. However, when cell growth was observed on plates having galactose as a carbon source as gene inducer, wild‐type Ate1 was found to be lethal for *ate1*∆ yeast cells, and the Ate1‐D277A mutation rescued the cells from Ate1‐mediated lethality (Fig. 4D). Moreover, the charge reversal at 277th position (D277K), completely disrupts the structure of Ate1 leading to loss of protein stability, enzyme activity and its function suggesting that aspartic acid is a key amino acid residue responsible for Ate1 functionality (Fig. S4, Table S4).

Taken together, our data suggest that the conserved cofactor‐binding cysteine cluster and active site residue D277 involved in active site formation and tRNA^Arg^ interaction, residing in the N‐terminal half of Ate1, are crucial for Ate1‐mediated cell death, which depends on Ate1 enzyme catalytic activity and stability, respectively.

### The C‐terminal domain is essential for the stability and catalytic activity of the Ate1 enzyme

**Fig. 5:**
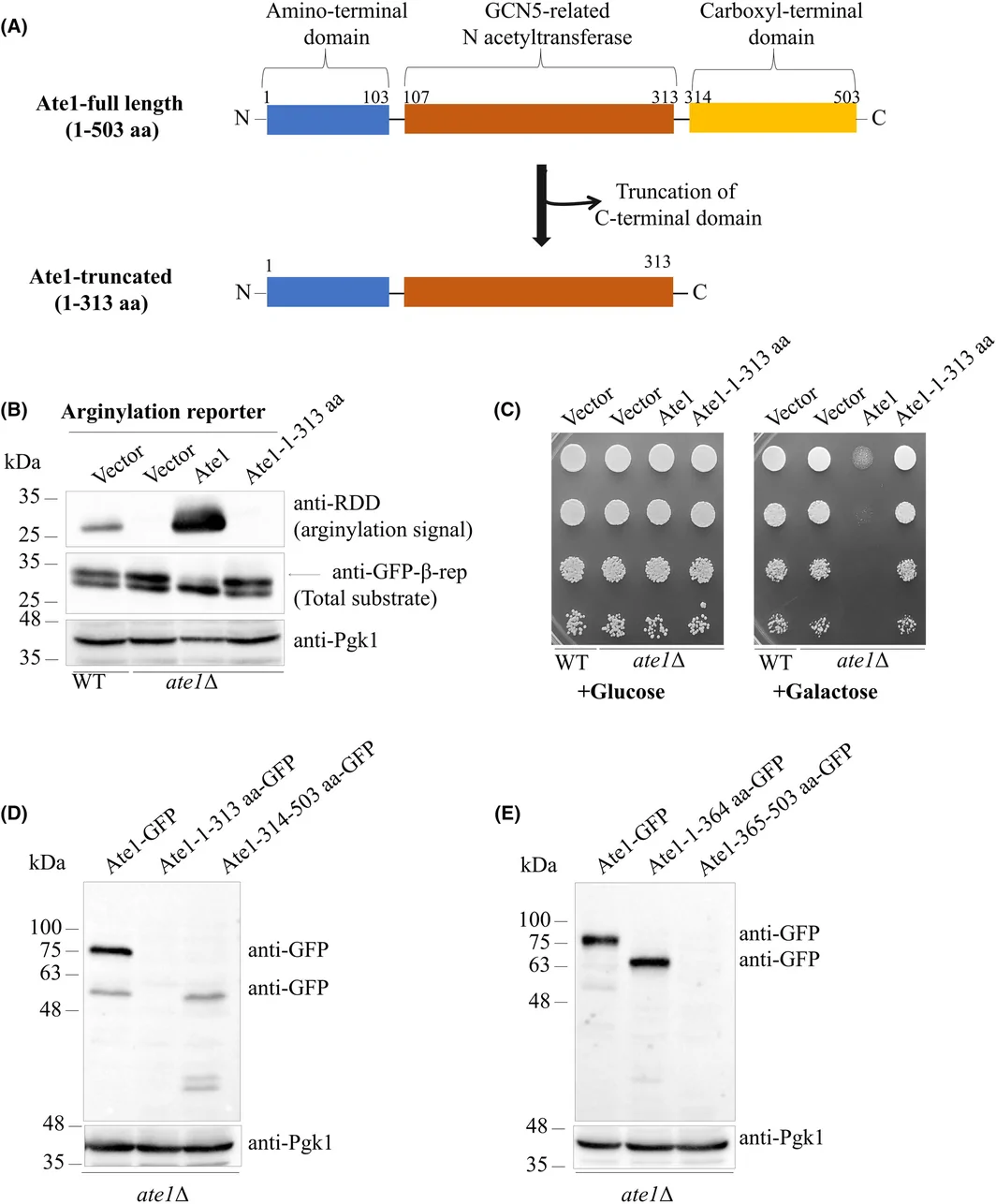
The C‐terminal domain is essential for Ate1‐mediated arginylation, cell death and protein stability. (A) The panel shows the domain architecture of full‐length Ate1 (Ate1‐1‐503 aa) and truncated Ate1 (Ate1‐1‐313 aa). (B) Immunoblot showing the arginylation and steady‐state level of arginylation reporter in *ate1*Δ yeast cells expressing the full‐length and truncated versions of Ate1 (Ate1‐1‐313 aa). The wild‐type yeast cells transformed with the vector act as a positive control, while the *ate1*Δ yeast cells transformed with the vector act as a negative control for this *in vitro* assay. Pgk1, a cytosolic enzyme, was used as a loading control. (C) Spotting assay showing the growth pattern of *ate1*Δ yeast cells transformed with the vector, full‐length Ate1 and truncated‐Ate1 (Ate1‐1‐313 aa) on SD‐glucose (left panel, repressible condition) and SD‐galactose (right panel, inducible condition) plates. Both wild‐type and truncated‐Ate1 were cloned under the galactose promoter. (D, E) The GFP‐tagged full‐length and truncated versions of Ate1 were expressed in *ate1*∆ cells. Immunoblot shows their stability, examined using an anti‐GFP antibody against the GFP tag present at the C terminus of Ate1.

Our structural and mutational analysis revealed that the cofactor‐binding cysteine cluster and active site residues present in the N‐terminal half of Ate1 are essential for enzyme activity and lethality. However, the essentiality of the Ate1 C‐terminal variable domain remains elusive. This prompted us to examine the importance of this domain in Ate1 activity. To investigate this, we constructed a truncated version of Ate1 by removing its C‐terminal half (Fig. 5A). We cloned the truncated Ate1 (Ate1‐1‐313 aa) under a galactose‐inducible promoter and expressed it in *ate1*Δ cells. First, we checked the arginylation activity by carrying out an *in vitro* arginylation assay and immunoblotting. We observed that the full‐length Ate1 arginylates the reporter substrate and leads to its reduced level in *ate1*Δ cells. However, the truncated Ate1 (Ate1‐1‐313 aa) failed to arginylate the substrate and does not affect the reporter stability (Fig. 5B, Table S5). Next, we performed a serial dilution‐based cell growth assay and found that the overexpression of full‐length Ate1 was lethal to the yeast, while the truncated Ate1 (Ate1‐1‐313 aa) rescued the cells from Ate1‐mediated cellular toxicity (Fig. 5C). The results confirmed that the C‐terminal domain is essential for Ate1 protein to carry out arginylation and for causing cell death.

Further, to determine whether this loss of function of Ate1 upon truncation is associated with protein stability, we tagged full‐length and truncated Ate1 versions (Ate1‐1‐313 aa and Ate1‐314‐503 aa) with GFP and performed immunoblotting. We found that the full‐length Ate1 was stable; however, the truncated Ate1 having the N‐terminal half (Ate1‐1‐313 aa) could not be detected. The truncated Ate1 (Ate1‐314‐503 aa) having the C‐terminal domain was detected but at a lower intensity than full‐length Ate1 (Fig. 5D, Table S5).

Interestingly, we noticed the presence of a protein band of relatively low molecular weight in the case of full‐length Ate1, whose size was similar to the protein band observed for truncated Ate1 (Ate1‐314‐503 aa), pointing to the presence of an enzyme cleavage site between the 300‐315 aa region of Ate1. Next, to identify the potential protease cleavage sites in the Ate1 protein sequence, we used the ExPASy Peptide Cutter tool. We found that Ate1 contains only one caspase cleavage site at the 364th position (Fig. S5). Thus, we cloned the region of Ate1 having 1–364 aa in the pYES‐Ate1‐GFP plasmid and checked its expression via immunoblotting. Surprisingly, we found that the truncated Ate1 with the N‐terminal domain (Ate1‐1‐364 aa) protein was stable and its expression was similar to full‐length Ate1. Moreover, the expression of the Ate1 C‐terminal domain, having 365–503 amino acids, was not detectable at the protein level, suggesting that the presence of 50 amino acid residues (Ate1‐314‐364 aa) of the C‐terminal domain is essential for Ate1 stability (Fig. 5E, Table S5). Overall, these findings show that the variable C‐terminal domain is indispensable for Ate1 stability, arginylation activity, as well as for Ate1‐mediated cell death.

### *In vivo* or *in vitro* co‐expression of N‐ and C‐terminal halves of Ate1 does not reconstitute Ate1 functionality

**Fig. 6:**
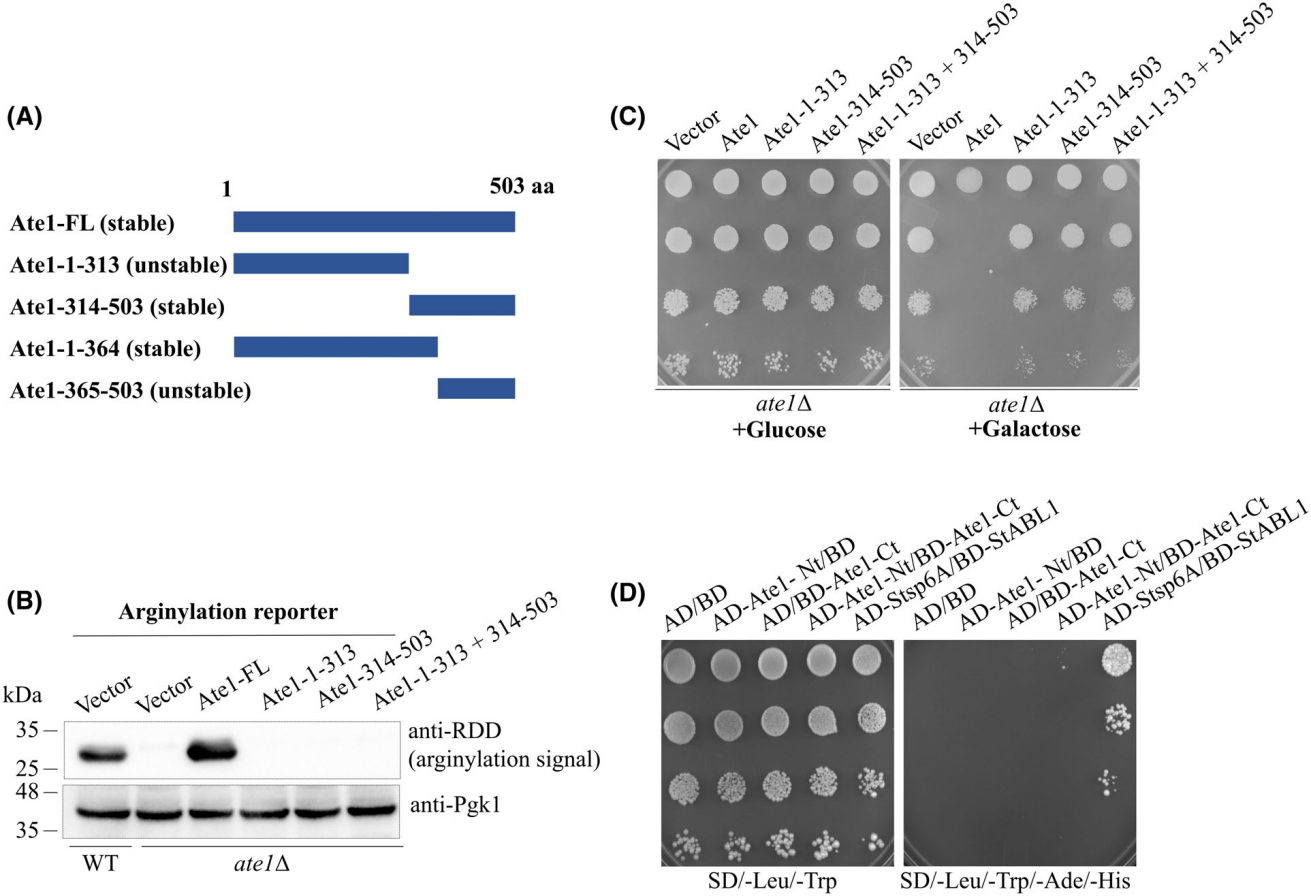
*In vivo* reconstitution of N‐terminal and C‐terminal halves of Ate1 did not restore Ate1 activity. (A) Schematic representation of the Ate1 protein and its truncated version. (B) Immunoblot showing the arginylation signal in the *ate1Δ* yeast cell expressing the full‐length Ate1 or its N‐ and C‐terminal halves with the indicated amino acid length. The WT and *ate1*Δ yeast cells transformed with the vector act as positive and negative controls, respectively. Pgk1, a cytosolic enzyme, was used as a loading control. (C) Spotting assay depicting the growth pattern of *ate1*Δ yeast cells carrying either vector, full‐length Ate1 or truncated Ate1‐1‐313 or Ate1‐314‐503 or both together on SD‐glucose (left panel, repressible condition) and SD‐galactose (right panel, inducible condition). (D) Yeast‐2 hybrid assay showing the lack of interaction between the N‐terminal and C‐terminal domains of Ate1. The AH109 cells transformed with different combinations of AD and BD vectors were serially diluted and grown on SD plates lacking ‐leu‐trp (permissive) and ‐leu‐trp‐ade‐his (selective). The cotransformed empty AD and BD vectors were used as negative controls, while the co‐transformed AD and BD vectors carrying Stsp6 and StABL1, respectively, were used as positive controls for the Y2H assay.

The Ate1 protein in yeast comprises three major domains whose truncation to various lengths deprived Ate1 of being stable, catalytically active and carrying out arginylation (Fig. 6A, Table S5). So, we tried to co‐express N‐terminal and C‐terminal halves *in vivo* and examined whether their co‐expression can restore arginylation and the lethal phenotype associated with Ate1. For this purpose, the halves were cloned into two different vectors under the Gal‐promoter (pRS423‐Gal‐Ate1 1‐313 aa, N‐terminal half and pYES‐Ate1‐314‐503 aa, C‐terminal half) and then co‐expressed in *ate1*Δ yeast cells. The cell lysate was prepared from the *ate1*Δ cells expressing either N‐terminal or C‐terminal halves or both and then mixed with the *ate1*Δ cells lysate expressing the arginylation reporter and incubated at 37 °C. After incubating the reaction mixture for 1 h, arginylation activity was evaluated by immunoblotting with an arginylation‐specific antibody as described above. We found that the *ate1*∆ yeast cells expressing either the N‐terminal half (Ate1‐1‐313 aa) or the C‐terminal half (Ate1‐314‐503 aa) of Ate1 or both together did not show arginylation activity, unlike the yeast cells expressing full‐length Ate1 (Fig. 6B, Table S5). Further, we investigated the impact of domain separation on Ate1 mediated cell death by serial dilution‐based growth assay and observed that the co‐expression of both these domains together was not lethal for yeast cells; however, expression of full‐length Ate1 killed yeast cells (Fig. 6C).

Since we observed that the N‐terminal half (Ate1‐1‐313 aa) was unstable, we tried to reconstitute the arginylation activity by co‐expressing the overlapping pairs containing stable N‐terminal half (Ate1‐1‐364 aa) with the stable C‐terminal half (Ate1‐314‐503 aa) of Ate1 in *ate1*Δ cells. We observed the presence of an arginylation signal in *ate1*Δ cells expressing full‐length Ate1 only and not in *ate1*Δ cells expressing either Ate1‐1‐364 or Ate1‐314‐503 alone or together (Fig. S6, Table S5). The lack of arginylation of the reporter substrate suggests that the co‐expression of stable truncated Ate1 versions, comprising the N‐ and C‐terminal halves of Ate1, failed to reconstitute the activity.

Further, to test whether the N‐terminal (Ate1‐1‐313 aa) and C‐terminal regions (Ate1‐314‐503 aa) of Ate1 physically interact upon separation, we cloned these domains into Y2H plasmids pGADT7 (having GAL activation domain) and pGBKT7 (having DNA‐binding domain), respectively, and cotransformed them into AH109 yeast strain. Next, we performed a serial dilution‐based growth assay on both permissive (‐leu‐trp) and selective media (‐leu‐trp‐ade‐his). Our results showed that the Ate1 N‐terminal and C‐terminal domains were not interacting upon separation, as yeast cells expressing them together were not able to grow in selective medium (Fig. 6D). This loss of interaction was expected as Ate1, having only the N‐terminal region, loses its stability upon truncation.

Overall, these data reveal that not only the stability but possibly the cotranslational folding of protein, as well as their proper domain orientation, are crucial for Ate1 catalytic activity.

### Mouse Ate1 is catalytically active; however, its overexpression is not lethal in yeast

**Fig. 7:**
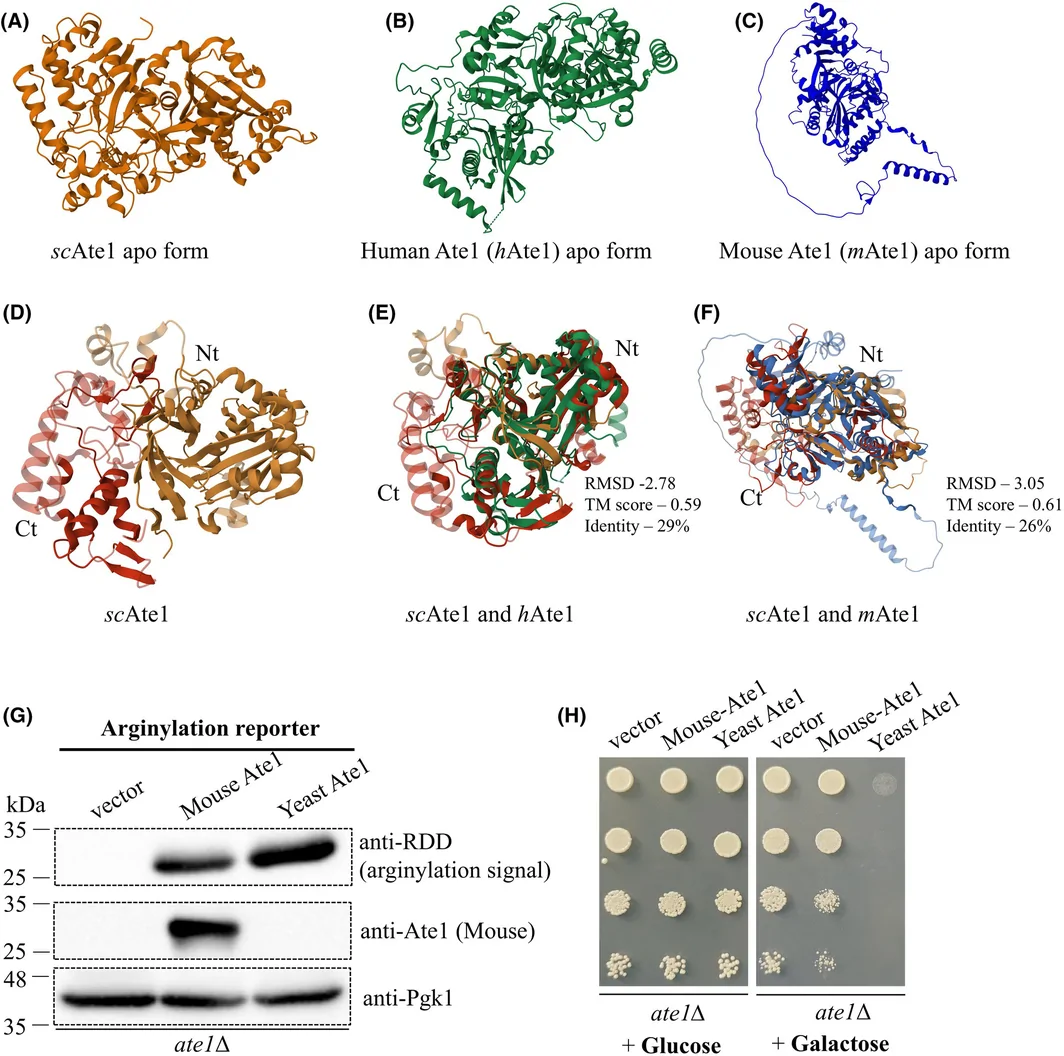
Comparative analysis between yeast, mouse and human Ate1. The three‐dimensional structure of Ate1 in apo form: (A) *Saccharomyces cerevisiae* Ate1 (*sc*Ate1), PDB 8E3S; (B) Human Ate1 (hAte1), PDB 8TZV; (C) Mouse Ate1, Alpha Fold ID: AF‐Q9Z2A5‐F1‐v6. The yeast and mouse Ate1 are monomeric proteins, while human Ate1 exists as a dimer. (D) *sc*Ate1 with highlighted C‐terminal domain (314–503 aa) in red colour. (E, F) Structural superimposition of yeast Ate1 (N‐terminal domain in yellow and C‐terminal with red) with human (green colour) and mouse Ate1 (blue colour). Dark colour shows the aligned residues while light colour represents nonaligned ones. (G) Immunoblot showing that mouse Ate1 is stable and arginylates the Ate1 substrate upon its expression in *ate1*∆ cells. The arginylation signal was detected using anti‐RDD and reporter stability with anti‐GFP antibody. The primary antibody anti‐Ate1 was used to detect the expression of mouse Ate1. Pgk1 acts as a loading control. (H) Spotting assay showing the growth phenotype of *ate1*∆ cells transformed with either vector, mouse Ate1 or yeast Ate1, on SD‐glucose (left panel, repressible condition) and SD‐galactose (right panel, inducible condition). Original source of the image: pdb_00008e3s, https://doi.org/10.2210/pdb8E3S/pdb; pdb_00008tzv, https://doi.org/10.2210/pdb8TZV/pdb; AF‐Q9Z2A5‐F1‐v6, https://alphafold.ebi.ac.uk/entry/AF‐Q9Z2A5‐4‐F1.

The yeast Ate1 shares 30.65% and 29.12% identity with mouse and human Ate1 at the sequence level, respectively. The structure of Ate1 has now been modelled and determined in human and mouse as well, which reveals that mammalian Ate1 is structurally more complex than yeast [34, 35, 48]. Compared with the human Ate1 apo state that forms a symmetric homodimer, the yeast and mouse Ate1 exist as monomers (Fig. 7A–C). Our data show that the C‐terminal domain is indispensable for Ate1 catalytic activity in yeast and is variable at the sequence level as well. Now, to know the similarities and differences between yeast and mammalian Ate1 at structure level, we chose the apo state of yeast Ate1 (Fig. 7D) and performed structure alignment with mammalian Ate1 to reveal the conserved structural features and alignment gaps present among them. The superimposition of yeast Ate1 (PDB 8E3S) with human Ate1 (PDB 8TZV) reveals that the aligned protein structures were 29% identical and have an RMSD value of 2.78 angstroms and a TM score of 0.59, suggesting strong structural similarity with moderate divergence. We observed that the GNAT fold was conserved, while the major alignment gap of around 55 amino acids (light red colour) was present in human Ate1 (Fig. 7E). These amino acid residues lie within the C‐terminal domain of yeast Ate1. Further, we superimposed the structure of yeast Ate1 with modelled mouse Ate1 (Alpha Fold ID: AF‐Q9Z2A5‐F1‐v6). The RMSD value between the yeast and mouse Ate1 protein structures was 3.05 angstroms with a 0.61 TM score, which indicates that the core protein structure is conserved but with notable structural differences (Fig. 7F). We observed the presence of two alignment gaps. One gap was present in mouse Ate1, corresponding to yeast, that is, the absence of around 55 amino acids that lie within the C‐terminal domain of yeast Ate1. Another gap was present in the yeast Ate1 GNAT fold; we observed that mouse Ate1 contains an intrinsically disordered region within the GNAT fold, which was absent in yeast Ate1. These structural finding suggests that the Ate1 protein structure has evolved significant variations, but still maintains the GNAT fold.

Next, to examine whether the mouse Ate1 functionally complements the yeast *ate1∆*, we cloned the mouse Ate1 (isoform 1) in pYES2 yeast expression vector and transformed it into the *ate1*∆ yeast strain lacking the endogenous Ate1 and performed an *in vitro* arginylation assay. We found the presence of an anti‐RDD signal in *ate1*∆ cells expressing yeast as well as mouse Ate1, which suggests that mouse Ate1 is active and can arginylate the reporter substrate within the yeast. The expression of mouse Ate1 was also checked using anti‐Ate1‐mouse antibody, and the presence of a single band corresponding to mouse Ate1 confirms its stable expression within the yeast. Pgk1, a cytosolic yeast enzyme, was used as a loading control (Fig. 7G). Next, we performed serial dilution growth assay to examine the impact of mouse *ATE1* overexpression on yeast growth. We observed that the *ate1*∆ cells transformed with either vector or mouse or yeast Ate1 show normal growth phenotype on SD‐glucose plates, which acts as a repressible condition for Ate1 expression. However, when *ate1*∆ cells were grown on SD‐galactose plates, which acts as an inducer for Ate1 gene expression, we found that the mouse Ate1 was nonlethal, unlike the yeast Ate1, and cells show a growth pattern similar to that of *ate1*∆ cells carrying the empty vector (Fig. 7H). Our results show that despite the structural complexity between these organisms, the basic arginylation machinery and mechanism are conserved; however, the physiological functions regulated by Ate1 from lower to higher eukaryotes may vary due to different substrate specificity and localisation of Ate1.

## Discussion

Arginyltransferase 1 (Ate1), a multifunctional enzyme conserved from yeast to humans, plays a pivotal role in diverse eukaryotic cellular processes by post‐translationally conjugating the basic arginine residue at the N‐terminal of proteins, hence regulating protein function and stability [6]. Despite the extensive research of Ate1 in biological processes and structural characterisation studies involving the N‐terminal and central GNTA fold domain, the significance of the C‐terminal domain of this enzyme remains elusive [30, 33]. Our study has revealed that the variable C‐terminal domain, despite being less conserved, is indispensable for Ate1 stability, arginylation and Ate1‐mediated cell death in yeast.

The Ate1 N‐terminal half comprises the conserved Zn/Fe‐S binding CXXC motif, tRNA^Arg^, substrate interacting residues and catalytic site. Our data show that the C20, a key residue of the conserved cysteine cluster, is essential for arginylation and Ate1‐mediated cell death and directly affects the enzymatic function rather than Ate1 stability. Specifically, we observed that C20 is more important than C23 as the mutation of C20 into serine alone or together with C23 led to the complete loss of arginylation. These findings are consistent with and strongly supported by previous studies, which showed that the CXXC motif residues C20 and C23 are essential for binding of the Fe‐S cluster, enzymatic activity and for suppression of Ate1‐mediated cell toxicity [7, 30].

The active site amino acid D277, which has shown to be involved in catalysis of Ate1‐mediated arginylation in prior studies, was found to be essential for the stability as well as catalytic activity of Ate1 in yeast. We observed that the mutation of D277 into lysine resulted in loss of enzyme stability and function may be due to the strong electrostatic perturbations in Ate1, while its mutation to alanine, a more conservative substitution of D277, affects enzyme activity and Ate1‐mediated cell death rather than protein stability. In earlier studies, the mutation of the D277 corresponding residue, E277 to lysine or alanine in *kl*Ate1, and E387 to alanine in *h*Ate1, also affects Ate1 enzymatic activity, which may possibly arise from compromised protein stability upon charge reversal and due to impaired enzymatic activity post mutation to alanine [32, 34].

Further, our research deciphered the importance of the C‐terminal domain of the Ate1 enzyme, which was not clear to date, as this domain is less constrained by evolutionary pressures and has more flexibility in its structure and function as compared to the N‐terminal domain. We found that the C‐terminal domain contributes to the protein stability and is indispensable for arginylation and Ate1‐mediated cell death. Our data show that the truncated Ate1 (Ate1‐1‐313 aa) lacking the C‐terminal domain (Ate1‐314‐503 aa) was unstable and inactive; however, the full‐length Ate1 comprising both the N‐terminal (Ate1‐1‐313 aa) and C‐terminal (Ate1‐314‐503 aa) domains was stable and active, exhibiting the enzymatic activity and toxicity associated with Ate1 overexpression. Moreover, we analysed that the Ate1 having only the C‐terminal domain (Ate1‐314‐503 aa) was stable, but the expression was low compared to the full‐length Ate1. Surprisingly, we also demonstrated that the extension of the N‐terminal region up to Ate1 364 aa restored the protein stability, suggesting that the 50 amino acid residues (Ate1‐314‐364 aa) of the C‐terminal domain are essential for Ate1 stability.

We also attempted to reconstitute functional activity *in vivo* by co‐expressing the N‐terminal and C‐terminal halves of Ate1, but the co‐expression failed to restore Ate1 enzymatic activity. Also, we tested the physical interaction between the two domains using the Y2H assay which reported a lack of interaction between the two domains under the given experimental conditions. The failure of Ate1 reconstitution and interaction in our study may arise due to the structural disruption and the loss of co‐translational protein folding upon separation of domains as truncated Ate1 carrying amino acids 1‐313 loses its stability upon removal of the C‐terminal domain. Further study is required to identify how these domains function cooperatively to make the enzyme stable and active. Our findings show that both the N and C‐terminal domains of Ate1 are essential for Ate1‐mediated arginylation. To find how these domains are conserved during evolution, we performed structural alignment of yeast Ate1 with human and mouse Ate1. The alignment shows the conservation of the GNAT fold and identifies a gap corresponding to a region that lies within the variable C‐terminal domain of yeast Ate1, suggesting the loss or divergence of the C‐terminal domain during evolution in higher organisms. Moreover, we found that mouse Ate1 was stable and catalytically active in yeast, suggesting that despite the structural complexity, mouse Ate1 can be folded correctly to retain its catalytic function in a yeast system. However, the heterologous overexpression of mouse Ate1 was not lethal in yeast, indicating that physiological functions regulated by Ate1 from lower to higher eukaryotes may vary due to different substrate specificity and subcellular localisation.

Overall, our research underscores the essentiality of the Ate1 C‐terminal domain and the structural interdependence among N and C‐terminal domains to obtain a full‐length polypeptide integrity, responsible for maintaining stability and functionality of the enzyme. Furthermore, our study provides clearer insights for the structural basis of arginylation, advances our knowledge of the regulatory functions of arginylation in cells and provides a basis for future research into the structure–function analysis of Ate1.

## Conflicts of interest

The authors declare no conflicts of interest.

## Author contributions

AK conceived and supervised the study. AK, VKY and SS designed the experiments. VKY and SS performed most of the experiments, while the arginylation assay was performed by VM. VKY, SS, VM and MM analysed and interpreted the data. VKY and SS wrote the manuscript, and AK edited and revised the manuscript. All authors read and approved the final version of the manuscript.

## Supporting information


**Data S1.** Sequencing results (FASTA sequence and corresponding chromatogram highlighting the mutation site).
**Table S1.** List of yeast strains used in this study.
**Table S2.** List of plasmids used in this study.
**Table S3.** List of primers used in this study.
**Table S4.** Stability and activity of Ate1 mutants.
**Table S5.** Stability and activity of Ate1 truncates.
**Fig. S1.** The image shows the two Pfam domains identified in the Ate1, namely ATE_N and ATE_C, respectively.
**Fig. S2.** Multiple sequence alignment performed using Clustal Omega shows that the C‐terminal half of Ate1 is less conserved among eukaryotes.
**Fig. S3.** Schematic representation of the *in vitro* arginylation reaction.
**Fig. S4.** Image showing the effect of charge reversal of active site residues of Ate1 on enzyme activity and Ate1‐mediated cell death.
**Fig. S5.** Image showing the presence of only one Caspase‐1 (Casp1) cleavage site at Asp364 in *sc*Ate1.
**Fig. S6.** Co‐expression of stable Ate1 truncation regions failed to reconstitute enzymatic activity *in vivo*.

## Data Availability

The data that support the findings of this study are available from the corresponding author upon reasonable request.

## References

[1] Manahan CO and App AA (1973) An Arginyl‐transfer ribonucleic acid protein transferase from cereal embryos. Plant Physiol 52, 13–16. 10.1104/pp.52.1.13 PMC366429 16658490

[2] Lock RA , Harding HWJ and Rogers GE (1976) Arginine transferase activity in homogenates from Guinea pig hair follicles. J Invest Dermatol 67, 582–586. 10.1111/1523-1747.ep12541685 977987

[3] Balzi E , Choder M , Chen W , Varshavsky A and Goffeau A (1990) Cloning and functional analysis of the arginyl‐tRNA‐protein transferase gene ATE1 of *Saccharomyces cerevisiae* . J Biol Chem 265, 7464–7471. 2185248

[4] Karakozova M , Kozak M , Wong CCL , Bailey AO , Iii JRY , Mogilner A , Zebroski H and Kashina A (2006) Arginylation of b‐actin regulates. Science 80, 192–196. 10.1126/science.1129344 16794040

[5] Kashina A (2014) Protein arginylation, a global biological regulator that targets actin cytoskeleton and the muscle. Anat Rec 297, 1630–1636. 10.1002/ar.22969 PMC4135399 25125176

[6] Galiano MR , Goitea VE and Hallak ME (2016) Post‐translational protein arginylation in the normal nervous system and in neurodegeneration. J Neurochem 1, 506–517. 10.1111/jnc.13708 27318192

[7] Kumar A , Birnbaum MD , Patel DM , Morgan WM , Singh J and Barrientos A (2016) Posttranslational arginylation enzyme Ate1 affects DNA mutagenesis by regulating stress response. Nat Publ gr 1–16. 10.1038/cddis.2016.284 PMC5059882 27685622

[8] Fina ME , Wang J , Vedula P , Tang HY , Kashina A and Dong DW (2022) Arginylation regulates G‐protein signaling in the retina. Front Cell Dev Biol 9, 1–14. 10.3389/fcell.2021.807345 PMC8815403 35127722

[9] Xu C , Li YM , Sun B , Zhong FJ and Yang LY (2021) ATE1 inhibits liver cancer progression through RGS5‐mediated suppression of Wnt/β‐catenin signaling. Mol Cancer Res 19, 1441–1453. 10.1158/1541-7786.MCR-21-0027 PMC9398136 34158395

[10] Soffer RL (1968) The arginine transfer reaction. Biochimica et Biophysica Acta (BBA) 155, 228–240. 10.1016/0005-2787(68)90352-3 5647059

[11] Varshavsky A (1992) The N‐end rule review. Cell 69, 725–735. 10.1016/0092-8674(92)90285-k 1317266

[12] Varshavsky A (2011) The N‐end rule pathway and regulation by proteolysis. Protein Sci 20, 1298–1345. 10.1002/pro.666 PMC3189519 21633985

[13] Kwon YT , Kashina AS , Davydov IV , Hu RG , An JY , Seo JW , Du F and Varshavsky A (2002) An essential role of N‐terminal arginylation in cardiovascular development. Science 297, 96–99. 10.1126/science.1069531 12098698

[14] Bachmair A , Finley D and Varshavsky A (1986) In vivo half‐life of a protein is a function of its amino‐terminal residue. Science 234, 179–186. 10.1126/science.3018930 3018930

[15] Gonda DK , Bachmair A , Wunning I , Tobias JW , Lane WS and Varshavsky A (1989) Universality and stucture of the N‐end rule. J Biol Chem 264, 16700–16712. 2506181

[16] Xie Y and Varshavsky A (1999) The E2‐E3 interaction in the N‐end rule pathway: The RING‐H2 finger of E3 is required for the synthesis of multiubiquitin chain. EMBO J 18, 6832–6844. 10.1093/emboj/18.23.6832 PMC1171746 10581257

[17] Chakraborty G (1993) N‐terminal Arginylation and ubiquitin‐ mediated proteolysis in nerve regeneration. Brain Res Bull 30, 439–445. 10.1016/0361-9230(93)90276-h 8384516

[18] Hallak ME and Bongiovanni G (1997) Posttranslational Arginylation of brain proteins. Neurochem Res 22, 467–473. 10.1023/a:1027315912242 9130258

[19] Davydov IV and Varshavsky A (2000) RGS4 is arginylated and degraded by the N‐end rule pathway in vitro. J Biol Chem 275, 22931–22941. 10.1074/jbc.M001605200 10783390

[20] Hwang CS , Shemorry A , Auerbach D and Varshavsky A (2010) The N‐end rule pathway is mediated by a complex of the RING‐type Ubr1 and HECT‐type Ufd4 ubiquitin ligases. Nat Cell Biol 12, 1177–1185. 10.1038/ncb2121 PMC3003441 21076411

[21] Chen L and Kashina A (2022) Arginylation regulates cytoskeleton organization and cell division and affects mitochondria in fission yeast. Mol Cell Biol 42, 1–14. 10.1128/mcb.00261-22 PMC9670973 36226970

[22] Rassier DE and Kashina A (2019) Protein arginylation of cytoskeletal proteins in the muscle : modifications modifying function. Am J Physiol Cell Physiol 316, 668–677. 10.1152/ajpcell.00500.2018 PMC6580163 30789755

[23] Lee MJ , Tasaki T , Moroi K , An JY , Kimura S , Davydov IV and Kwon YT (2005) RGS4 and RGS5 are in vivo of the N‐end rule pathway. Proc Natl Acad Sci USA 102, 15030–15035. 10.1073/pnas.0507533102 PMC1257735 16217033

[24] Fina ME , Wang J , Nikonov SS , Sterling S , Vardi N , Kashina A and Dong DW (2021) Arginyltransferase (Ate1) regulates the RGS7 protein level and the sensitivity of light‐evoked ON‐bipolar responses. Sci Rep 11, 1–20. 10.1038/s41598-021-88628-3 PMC8087773 33931669

[25] Bongiovanni G , Fidelio GD , Barra HS and Hallak ME (1995) The post‐translational incorporation of arginine into a β‐amyloid peptide increases the probability of α‐helix formation. Neuroreport 7, 326–328. 8742481

[26] Wang J , Han X , Leu NA , Sterling S , Kurosaka S , Fina M , Lee VM , Dong DW , Iii JRY and Kashina A (2017) Protein arginylation targets alpha synuclein, facilitates normal brain health, and prevents neurodegeneration. Sci Rep 7, 1–14. 10.1038/s41598-017-11713-z PMC5595787 28900170

[27] Lazar I , Fabre B , Feng Y , Khateb A , Frit P , Kashina A , Zhang T , Avitan‐Hersh E , Kim H , Brown K *et al*. (2022) Arginyl‐tRNA‐protein transferase 1 (ATE1) promotes melanoma cell growth and migration. FEBS Lett 596, 1468–1480. 10.1002/1873-3468.14376 PMC10118390 35561126

[28] Kashina AS (2015) Chapter 1 Protein Arginylation : Over 50 Years of Discovery. Methods Mol Biol 1337, 1–11. 10.1007/978-1-4939-2935-1_1 26285874

[29] Moorthy BT , Jiang C , Patel DM , Ban Y , O'Shea CR , Kumar A , Yuan T , Birnbaum MD , Gomes AV , Chen X *et al*. (2022) The evolutionarily conserved arginyltransferase 1 mediates a pVHL‐independent oxygen‐sensing pathway in mammalian cells. Dev Cell 57, 654–669. 10.1016/j.devcel.2022.02.010 PMC8957288 35247316

[30] Van V , Brown JB , O'Shea CR , Rosenbach H , Mohamed I , Ejimogu NE , Bui TS , Szalai VA , Chacón KN , Span I *et al*. (2023) Iron‐sulfur clusters are involved in post‐translational arginylation. Nat Commun 14, 458. 10.1038/s41467-023-36158-z PMC9884297 36709327

[31] Kumar A , O'Shea CR , Yadav VK , Kandasamy G , Moorthy BT , Ambrose EA , Mulati A , Fontanesi F and Zhang F (2025) Arginyltransferase1 drives a mitochondria‐dependent program to induce cell death. Cell Death Dis 16, 622. 10.1038/s41419-025-07917-1 PMC12357888 40818972

[32] Heon B , Kyung M , Joo S , The K , Hoe J and Varshavsky A (2022) Crystal structure of the Ate1 arginyl‐tRNA‐protein transferase and arginylation of N‐degron substrates. Proc Natl Acad Sci USA 119, 1–11. 10.1073/pnas.2209597119 PMC9351520 35878037

[33] Abeywansha T , Huang W , Ye X , Nawrocki A , Lan X , Jankowsky E , Taylor DJ and Zhang Y (2023) The structural basis of tRNA recognition by arginyl‐tRNA‐protein transferase. Nat Commun 14, 2232. 10.1038/s41467-023-38004-8 PMC10115844 37076488

[34] Lan X , Huang W , Kim SB , Fu D , Abeywansha T , Lou J , Balamurugan U , Kwon YT , Ji CH , Taylor DJ *et al*. (2024) Oligomerization and a distinct tRNA‐binding loop are important regulators of human arginyl‐transferase function. Nat Commun 15, 6350. 10.1038/s41467-024-50719-w PMC11283454 39068213

[35] Van V , Ejimogu NE , Bui TS and Smith AT (2022) The structure of Saccharomyces cerevisiae Arginyltransferase 1 (ATE1). J Mol Biol 434, 167816. 10.1016/j.jmb.2022.167816 PMC9992452 36087779

[36] Rai R , Mushegian A , Makarova K and Kashina A (2006) Molecular dissection of arginyltransferases guided by similarity to bacterial peptidoglycan synthases. EMBO Rep 7, 800–805. 10.1038/sj.embor.7400747 PMC1525158 16826240

[37] Momose K and Kaji A (1966) Soluble amino acid‐incorporating system. J Biol Chem 241, 3294–3307. 5330431

[38] Hegde SS and Shrader TE (2001) FemABX family members are novel nonribosomal Peptidyltransferases and important pathogen‐specific drug targets. J Biol Chem 276, 6998–7003. 10.1074/jbc.M008591200 11083873

[39] Suto K , Shimizu Y , Watanabe K , Ueda T , Fukai S , Nureki O and Tomita K (2006) Crystal structures of leucyl/phenylalanyl‐tRNA‐protein transferase and its complex with an aminoacyl‐tRNA analog. EMBO J 25, 5942–5950. 10.1038/sj.emboj.7601433 PMC1698881 17110926

[40] Dempsey LA (2011) No regulation. Nat Immunol 12, 931.

[41] Ud‐Din AIMS , Tikhomirova A and Roujeinikova A (2016) Structure and functional diversity of GCN5‐related n‐acetyltransferases (GNAT). Int J Mol Sci 17, 1018. 10.3390/ijms17071018 PMC4964394 27367672

[42] Friedrich UA , Zedan M , Hessling B , Fenzl K , Gillet L , Barry J , Knop M , Kramer G and Bukau B (2021) Nα‐terminal acetylation of proteins by NatA and NatB serves distinct physiological roles in Saccharomyces cerevisiae. Cell Rep 34, 108711. 10.1016/j.celrep.2021.108711 33535049

[43] Macwilliams MP and Liao M (2006) Luria broth (LB) and Luria agar (LA) media and their uses protocol. *Am Soc Microbiol*.

[44] Bergman LW (2001) Growth and maintenance of yeast. Methods Mol Biol 177, 9–14. 10.1385/1-59259-210-4:009 11530618

[45] Hanahan D (1983) Studies on transformation of Escherichia coli with plasmids. J Mol Biol 166, 557–580. 10.1016/s0022-2836(83)80284-8 6345791

[46] Agatep R , Kirkpatrick RD , Parchaliuk DL , Woods RA and Gietz RD (1998) Transformation of Saccharomyces cerevisiae by the lithium acetate/single‐stranded carrier DNA/polyethylene glycol protocol. Tech Tips Online 3, 133–137.

[47] Kumar A and Zhang F (2023) Assaying Arginylation activity in cell lysates using a fluorescent reporter. Methods in Molecular Biology 2620, 71–80. 10.1007/978-1-0716-2942-0_9 37010750

[48] Ate A , Cartwright M , Parakra R , Oduwole A , Zhang F , Deredge DJ and Smith AT (2024) Identification of an intrinsically disordered region (IDR) in Arginyltransferase 1 (ATE1). Biochemistry 63, 3236–3249. 10.1021/acs.biochem.4c00512 PMC12045025 39642180

