# Supplementary material for: The C‐terminal domain of yeast Arginyltransferase1 is essential for its catalytic activity
Source: FEBS Open Bio. 2026 Sep 21:10.1002/2211-5463.70336. Online ahead of print. doi: 10.1002/2211-5463.70336 (PMC13592012; doi:10.1002/2211-5463.70336)
Supplement: Supplementary file 1 [file FEB4-9999-0-s001.docx]

**Supplementary File**

**The C-terminal domain of yeast Arginyltransferase1 (Ate1) is essential for its catalytic activity**

Vikas Kumar Yadav^1*^, Shweta^1*^, Manisha Malhotra^1^, Vikash Maurya^1^ and Akhilesh Kumar^1†^

**Supplementary Data 1**: **Sequencing results (FASTA sequence and corresponding chromatogram highlighting the mutation site)**

**1. Ate1-C20S mutant**

**>PYES-20S-GFP_ATE1-SEQ-200BP-R-_C02.ab1**

TTTGGTTTCAATACCAGGCCGGGCTTACCACAAGAAAAGGTAGGAATTAATAAAGACTGCAAAACCAATGATATTAAGTATGAATATATGTGACCCCCATTATTACCGTGACAATACCCAGACTTTGCGGCAGGTTCATTGTGCATCGAGGGAGCCCAAATAACGAATCTATCGGACATGGCGGTACCAAGCTTAATATTCCCTATAGTGAGTCGTATTACAGCTGCTAGTAGTCCGATCCGGGGTTTTTTCTCCTTGACGTTAAAGTATAGAGGTATATTAACAATTTTTTGTTGATACTTTTATTACATTTGAATAAGAAGTAATACAAACCGAAAATGTTGAAAGTATTAGTTAAAGTGGTTATGCAGTTTTTGCATTTATATATCTGTTAATAGATCAAAAATCATCGCTTCGCTGATTAATTACCCCAGAAATAAGGCTAAAAAACTAATCGCATTATCATCCTATGGTTGTTAATTTGATTCGTTCATTTGAAGGTTTGTGGGGCCAGGTTACTGCCAATTTTTCCTCTTCATAACCATAAAAGCTAGTATTGTAGAATCTTTATTGTTCGGAGCAGTGCGGCGCGAGGCACATCTGCGTTTCAGGAACGCGACCGGTGAAGACGAGGACGCACGGAGGAGAGTCTTCCTTCGGAGGGCTGACACCCGCTCGGCGGCTTCTAATCCGAGGACTAATGGATCAATCCACAAGATCCCTGTA

**>PYES-20S-GFP_ATE1-SEQ-200BP-R-_C02.ab1 (Reverse and Complement)**

TACAGGGATCTTGTGGATTGATCCATTAGTCCTCGGATTAGAAGCCGCCGAGCGGGTGTCAGCCCTCCGAAGGAAGACTCTCCTCCGTGCGTCCTCGTCTTCACCGGTCGCGTTCCTGAAACGCAGATGTGCCTCGCGCCGCACTGCTCCGAACAATAAAGATTCTACAATACTAGCTTTTATGGTTATGAAGAGGAAAAATTGGCAGTAACCTGGCCCCACAAACCTTCAAATGAACGAATCAAATTAACAACCATAGGATGATAATGCGATTAGTTTTTTAGCCTTATTTCTGGGGTAATTAATCAGCGAAGCGATGATTTTTGATCTATTAACAGATATATAAATGCAAAAACTGCATAACCACTTTAACTAATACTTTCAACATTTTCGGTTTGTATTACTTCTTATTCAAATGTAATAAAAGTATCAACAAAAAATTGTTAATATACCTCTATACTTTAACGTCAAGGAGAAAAAACCCCGGATCGGACTACTAGCAGCTGTAATACGACTCACTATAGGGAATATTAAGCTTGGTACCGCCATGTCCGATAGATTCGTTATTTGGGCTCCCTCGATGCACAATGAACCTGCCGCAAAGTCTGGGTATTGTCACGGTAATAATGGGGGTCACATATATTCATACTTAATATCATTGGTTTTGCAGTCTTTATTAATTCCTACCTTTTCTTGTGGTAAGCCCGGCCTGGTATTGAAACCAAA

TCGATGCACAATGAACCTGCCGCAAAGTGTGGGTATTGTCACGGTAATAAGGGGGGC **WT-ATE1**

TCGATGCACAATGAACCTGCCGCAAAGTCTGGGTATTGTCACGGTAATAATGGGGGT **ATE1-C20S (R & C)**

ACCCCCATTATTACCGTGACAATACCCAGACTTTGCGGCAGGTTCATTGTGCATCGA **ATE1-C20S (reverse Sanger sequencing read)**

**2. Ate1-C23S mutant**

**>PYES-23S-GFP_ATE1-SEQ-200BP-R-_F06.ab1**

TTTCATCTTTACAACGTACCATATTTGTTCATATAACGATGAGCCCACGAATCCAATGCGACCTACTGATCCATGTTGCCCCCCTTATTACCGTGAGAATACCCACACTTTGCGGCAGGTTCATTGTGCATCGAGGGAGCCCAAATAACGAATCTATCGGACATGGCGGTACCAAGCTCAATATTCCCTATAGTGAGTCGTATTACAGCTGCTAGTAGTCCGATCCGGGGTTTTTTCTCCTTGACGTTAAAGTATAGAGGTATATTAACAATTTTTTGTTGATACTTTTATTACATTTGAATAAGAAGTAATACAAACCGAAAATGTTGAAAGTATTAGTTAAAGTGGTTATGCAGTTTTTGCATTTATATATCTGTTAATAGATCAAAAATCATCGCTTCGCTGATTAATTACCCCAGAAATAAGGCTAAAAAACTAATCGCATTATCATCCTATGGTTGTTAATTTGATTCGTTCATTTGAAGGTTTGTGGGGCCAGGTTACTGCCAATTTTTCCTCTTCATAACCATAAAAGCTAGTATTGTAGAATCTTTATTGTTCGGAGCAGTGCGGCGCGAGGCACATCTGCGTTTCAGGAACGCGACCGGTGAAAACAAGGACGCACGGAGGAGAGTCTTCCTTCGGAGGGCTGTCACCCGCTCGGCGGCTTCTAATCCGTGGACTAATGGATCGATCCCACGCGCCCTGTAGCGGCACTTAAACCCGGCAGGTGAAGAGTAAACACCCAAGAAACAACACACAAGAAAAGCCAAAAAAGCAAGCCACATGCCAGAAAT

**>PYES-23S-GFP_ATE1-SEQ-200BP-R-_F06.ab1 (Reverse and Complement)**

ATTTCTGGCATGTGGCTTGCTTTTTTGGCTTTTCTTGTGTGTTGTTTCTTGGGTGTTTACTCTTCACCTGCCGGGTTTAAGTGCCGCTACAGGGCGCGTGGGATCGATCCATTAGTCCACGGATTAGAAGCCGCCGAGCGGGTGACAGCCCTCCGAAGGAAGACTCTCCTCCGTGCGTCCTTGTTTTCACCGGTCGCGTTCCTGAAACGCAGATGTGCCTCGCGCCGCACTGCTCCGAACAATAAAGATTCTACAATACTAGCTTTTATGGTTATGAAGAGGAAAAATTGGCAGTAACCTGGCCCCACAAACCTTCAAATGAACGAATCAAATTAACAACCATAGGATGATAATGCGATTAGTTTTTTAGCCTTATTTCTGGGGTAATTAATCAGCGAAGCGATGATTTTTGATCTATTAACAGATATATAAATGCAAAAACTGCATAACCACTTTAACTAATACTTTCAACATTTTCGGTTTGTATTACTTCTTATTCAAATGTAATAAAAGTATCAACAAAAAATTGTTAATATACCTCTATACTTTAACGTCAAGGAGAAAAAACCCCGGATCGGACTACTAGCAGCTGTAATACGACTCACTATAGGGAATATTGAGCTTGGTACCGCCATGTCCGATAGATTCGTTATTTGGGCTCCCTCGATGCACAATGAACCTGCCGCAAAGTGTGGGTATTCTCACGGTAATAAGGGGGGCAACATGGATCAGTAGGTCGCATTGGATTCGTGGGCTCATCGTTATATGAACAAATATGGTACGTTGTAAAGATGAAA

AATGAACCTGCCGCAAAGTGTGGGTATTGTCACGGTAATAAGGGGGGCAACATGGAT **WT-ATE1**

AATGAACCTGCCGCAAAGTGTGGGTATTCTCACGGTAATAAGGGGGGCAACATGGAT **ATE1-C23S (R & C)**

ATCCATGTTGCCCCCCTTATTACCGTGAGAATACCCACACTTTGCGGCAGGTTCATT **ATE1-C23S (reverse Sanger sequencing read)**

**3. Ate1-C20,23S mutant**

**>PYES-20-23S-GFP_ATE1-SEQ-200BP-R-_G06.ab1**

TTTAATCTTTACAACGTCCATTTATGTTCATATAACGATGAGCCCACGAATCCAATGCGAAACAACTGATCCATGTTGCCCCCCTTATTACCGTGAGAATACCCAGACTTTGCGGCAGGTTCATTGTGCATCGAGGGAGCCCAAATAACGAATCTATCGGACATGGCGGTACCAAGCTTAATATTCCCTATAGTGAGTCGTATTACAGCTGCTAGTAGTCCGATCCGGGGTTTTTTCTCCTTGACGTTAAAGTATAGAGGTATATTAACAATTTTTTGTTGATACTTTTATTACATTTGAATAAGAAGTAATACAAACCGAAAATGTTGAAAGTATTAGTTAAAGTGGTTATGCAGTTTTTGCATTTATATATCTGTTAATAGATCAAAAATCATCGCTTCGCTGATTAATTACCCCAGAAATAAGGCTAAAAAACTAATCGCATTATCATCCTATGGTTGTTAATTTGATTCGTTCATTTGAAGGTTTGTGGGGCCAGGTTACTGCCAATTTTTCCTCTTCATAACCATAAAAGCTAGTATTGTAGAATCTTTATTGTTCGGAGCAGTGCGGCGCGAGGCACATCTGCGTTTCAGGAACGCGACCGGTGAAGACGAGGACGCACGGAGGAGAGTCTTCCTTCGGAGGGCTGTCACCCGCTCGGCGGCTTCTAATCCGTGGACTAGTGGATCGATCCCACGCGCCCTGTAGCGGCGCATT

AAGCGCCGGCGGGTGTGGTGGT

**>PYES-20-23S-GFP_ATE1-SEQ-200BP-R-_G06.ab1 (Reverse and Complement)**

ACCACCACACCCGCCGGCGCTTAATGCGCCGCTACAGGGCGCGTGGGATCGATCCACTAGTCCACGGATTAGAAGCCGCCGAGCGGGTGACAGCCCTCCGAAGGAAGACTCTCCTCCGTGCGTCCTCGTCTTCACCGGTCGCGTTCCTGAAACGCAGATGTGCCTCGCGCCGCACTGCTCCGAACAATAAAGATTCTACAATACTAGCTTTTATGGTTATGAAGAGGAAAAATTGGCAGTAACCTGGCCCCACAAACCTTCAAATGAACGAATCAAATTAACAACCATAGGATGATAATGCGATTAGTTTTTTAGCCTTATTTCTGGGGTAATTAATCAGCGAAGCGATGATTTTTGATCTATTAACAGATATATAAATGCAAAAACTGCATAACCACTTTAACTAATACTTTCAACATTTTCGGTTTGTATTACTTCTTATTCAAATGTAATAAAAGTATCAACAAAAAATTGTTAATATACCTCTATACTTTAACGTCAAGGAGAAAAAACCCCGGATCGGACTACTAGCAGCTGTAATACGACTCACTATAGGGAATATTAAGCTTGGTACCGCCATGTCCGATAGATTCGTTATTTGGGCTCCCTCGATGCACAATGAACCTGCCGCAAAGTCTGGGTATTCTCACGGTAATAAGGGGGGCAACATGGATCAGTTGTTTCGCATTGGATTCGTGGGCTCATCGTTATATGAACATAAATGGACGTTGTAAAGATTAAA

AATGAACCTGCCGCAAAGTGTGGGTATTGTCACGGTAATAAGGGGGGCAACATGGAT **WT-ATE1**

AATGAACCTGCCGCAAAGTCTGGGTATTCTCACGGTAATAAGGGGGGCAACATGGAT **ATE1-C20, C23 (R & C)**

ATCCATGTTGCCCCCCTTATTACCGTGAGAATACCCAGACTTTGCGGCAGGTTCATT **ATE1-C20, C23 (reverse Sanger sequencing read)**

**4. Ate1-D277A mutant**

**>0626_058_004_PLD_PLD4-D277A-COL-2_Ate1-SacI-R_E12.ab1**

CCTAGAAATCATATCCTGAATGGGTTTCAATGGGGATATATTTACTGTGACACACATCCAGTACTTCCGCACCATAGTTTGCTTTATAATTCATCTTCGGACAGTCCTCAATATAGTACCCCAGGTAATAGTATTGCAGATTTGTCCTTTGAATGATGGCAAGAGCGCGCAAAGCACTGAGCTTCCCTAGGGACCATTTACTGTAGTCTGGATCCCAAATAAAATATACCGAAGATATACCACTGGGCAAGATATCTGACACAGTGATGGCAATTAATTTACCCTCATAGTAGTAGCATTCGTGCACAGGACCCATATGCTTCAGTTTCTCGCCTGGCTTCATTCGCTGCCAATTGTTTAACTGCTCCCATGATTCTTGTGTCCCCAACACCGCTTCAGGGCCAAAGGGCGTATCACATAGGAATCGCTTAAAGGACTTCGGCGAATTATTGTAGTCTTGATGCACTTTTTCCTGATATTTGACAAATAAGTGATACTTTTCTTCACTATAAAGTGCAGGCTCAAATCGGGTATAGAATGTTTTAGAATTCATCTCTGCATTAACAATTTTGCCCACAAAATCACTGCTTGCAACAGCGGCTGGGCAGTAGTCCTCACTTGTTATTCTAGTAGCAAAGCGGCTGATGCATTTTTTCAATTCCTTTGTCATATTCAATTCTTGAGGGGCAGTTCGAATGGTGTATAACCTACAACAATTCCTCAACGGGTCCACTTTATAAAGAAACTTACCAGACCTTCTAAATCCCATATTACACATGCGATCATACGTGGCTACATCCATATGTTCCACGAAAGAGCCAATTGTGCAGTTTTCAATCTTTACAACGTCCATTTTGTTCATATAACGATGAGCCCACGAATCCAATGCGAACAACTGATCCATGTTGCCCCCCTTATTACCGTGACAATACCCACACTTTGCGGCAGGTTCATTGTGCATCGAGGGAGCCCAAATAACGAATCTATCGGACATGGCGGTACCAAGCTTAATATTCCCTATAGTGAGTCGTATTACAGCTGCTAGTAGTCCGATCCGGGGTTTTTTCTCCTTGACGTTAAAGTATAGAGGTTATTAACAATTTTTTGGTGATACTTTATTACATTTGAATAAGAGGAATACAACCCGAAAAGTTGAAAGATTAGTTAAAGGGGTATGCAGTTTTGCATTTAAATTCGGTAAAAGAAAAAAACATCGCTTCCCGGATAATTCCCCCAGAAAAGGGTAAAAACAATCCCCTTTACCCCCAGGGGGTTAATTGTTCCCTTTATTGAGGGTGTGGGGGGCGGCGTTCGCAATTTCTCTTCTCAAACCAAAAAAATAAAATTAAAAATTTTTTTTGGAAAAGGGGGCGGGGGGGACTTTGTTTTTAAAACACCGCGAGAAAAAACACAGGAAAAAATTTTTTTGGGGTCCCCCCCGGGGTTTTTTGGGATAAGGAACCCCCCCCCCCGCGCCGGATAAAAGGGGGGGGGGGGGGGGGGAGGCAAATTTTCCTCCCCCCCTT

**>0626_058_004_PLD_PLD4-D277A-COL-2_Ate1-SacI-R_E12.ab1 (Reverse and complement)**

AAGGGGGGGAGGAAAATTTGCCTCCCCCCCCCCCCCCCCCCTTTTATCCGGCGCGGGGGGGGGGGTTCCTTATCCCAAAAAACCCCGGGGGGGACCCCAAAAAAATTTTTTCCTGTGTTTTTTCTCGCGGTGTTTTAAAAACAAAGTCCCCCCCGCCCCCTTTTCCAAAAAAAATTTTTAATTTTATTTTTTTGGTTTGAGAAGAGAAATTGCGAACGCCGCCCCCCACACCCTCAATAAAGGGAACAATTAACCCCCTGGGGGTAAAGGGGATTGTTTTTACCCTTTTCTGGGGGAATTATCCGGGAAGCGATGTTTTTTTCTTTTACCGAATTTAAATGCAAAACTGCATACCCCTTTAACTAATCTTTCAACTTTTCGGGTTGTATTCCTCTTATTCAAATGTAATAAAGTATCACCAAAAAATTGTTAATAACCTCTATACTTTAACGTCAAGGAGAAAAAACCCCGGATCGGACTACTAGCAGCTGTAATACGACTCACTATAGGGAATATTAAGCTTGGTACCGCCATGTCCGATAGATTCGTTATTTGGGCTCCCTCGATGCACAATGAACCTGCCGCAAAGTGTGGGTATTGTCACGGTAATAAGGGGGGCAACATGGATCAGTTGTTCGCATTGGATTCGTGGGCTCATCGTTATATGAACAAAATGGACGTTGTAAAGATTGAAAACTGCACAATTGGCTCTTTCGTGGAACATATGGATGTAGCCACGTATGATCGCATGTGTAATATGGGATTTAGAAGGTCTGGTAAGTTTCTTTATAAAGTGGACCCGTTGAGGAATTGTTGTAGGTTATACACCATTCGAACTGCCCCTCAAGAATTGAATATGACAAAGGAATTGAAAAAATGCATCAGCCGCTTTGCTACTAGAATAACAAGTGAGGACTACTGCCCAGCCGCTGTTGCAAGCAGTGATTTTGTGGGCAAAATTGTTAATGCAGAGATGAATTCTAAAACATTCTATACCCGATTTGAGCCTGCACTTTATAGTGAAGAAAAGTATCACTTATTTGTCAAATATCAGGAAAAAGTGCATCAAGACTACAATAATTCGCCGAAGTCCTTTAAGCGATTCCTATGTGATACGCCCTTTGGCCCTGAAGCGGTGTTGGGGACACAAGAATCATGGGAGCAGTTAAACAATTGGCAGCGAATGAAGCCAGGCGAGAAACTGAAGCATATGGGTCCTGTGCACGAATGCTACTACTATGAGGGTAAATTAATTGCCATCACTGTGTCAGATATCTTGCCCAGTGGTATATCTTCGGTATATTTTATTTGGGATCCAGACTACAGTAAATGGTCCCTAGGGAAGCTCAGTGCTTTGCGCGCTCTTGCCATCATTCAAAGGACAAATCTGCAATACTATTACCTGGGGTACTATATTGAGGACTGTCCGAAGATGAATTATAAAGCAAACTATGGTGCGGAAGTACTGGATGTGTGTCACAGTAAATATATCCCCATTGAAACCCATTCAGGATATGATTTCTAGG

TCCCTAGGGAAGCTCAGTGCTTTGCGCGATCTTGCCATCATTCAAAGGACAAATCTG **WT-ATE1**

TCCCTAGGGAAGCTCAGTGCTTTGCGCGCTCTTGCCATCATTCAAAGGACAAATCTG **ATE1-D277A(R & C)**

GATTTGTCCTTTGAATGATGGCAAGAGCGCGCAAAGCACTGAGCTTCCCTAG **ATE1-D277A (reverse Sanger sequencing read)**

**5. Ate1-D277K mutant**

**>AKE-67-ATE1-D277K-GFP_Ate1-Sac1-R__A07.ab1**

TCTAGAAATCATATCCCTGAATGGGTTTCAATGGGTATATATTTACTGCCGACACACATCCAGTACTTCCGCACCATAGTTTGCTTTATAATTCATCTTCGGACAGTCCTCAATATAGTACCCCAGGTAATAGTATTGCAGATTTGTCCTTTGAATGATGGCAAGTTTGCGCAAAGCACTGAGCTTCCCTAGGGACCATTTACTGTAGTCTGGATCCCAAATAAAATATACCGAAGATATACCACTGGGCAAGATATCTGACACAGTGATGGCAATTAATTTACCCTCATAGTAGTAGCATTCGTGCACAGGACCCATATGCTTCAGTTTCTCGCCTGGCTTCATTCGCTGCCAATTGTTTAACTGCTCCCATGATTCTTGTGTCCCCAACACCGCTTCAGGGCCAAAGGGCGTATCACATAGGAATCGCTTAAAGGACTTCGGCGAATTATTGTAGTCTTGATGCACTTTTTCCTGATATTTGACAAATAAGTGATACTTTTCTTCACTATAAAGTGCAGGCTCAAATCGGGTATAGAATGTTTTAGAATTCATCTCTGCATTAACAATTTTGCCCACAAAATCACTGCTTGCAACAGCGGCTGGGCAGTAGTCCTCACTTGTTATTCTAGTAGCAAAGCGGCTGATGCATTTTTTCAATTCCTTTGTCATATTCAATTCTTGAGGGGCAGTTCTAATGGTGTATAACCTACAACCATTCCTCGACCGGTCCACTTTATAAGAAACTTACAGGACGTTCAAATCCGCTATTACACATGCGATCATAGAGTGGAACAATACATATGTGCAAGAAAAGACAATTGTGCGGTGTTCAATCTTTTCAAGTCCTTTTTGTGCATATAACCATGATCCCACCGAACAAATGAGAGCAACTGATCCCGTTAGCCCCCTTATAGTCGTGACAA

**>AKE-67-ATE1-D277K-GFP_Ate1-Sac1-R__A07.ab1 (Reverse and Complement)**

TTGTCACGACTATAAGGGGGCTAACGGGATCAGTTGCTCTCATTTGTTCGGTGGGATCATGGTTATATGCACAAAAAGGACTTGAAAAGATTGAACACCGCACAATTGTCTTTTCTTGCACATATGTATTGTTCCACTCTATGATCGCATGTGTAATAGCGGATTTGAACGTCCTGTAAGTTTCTTATAAAGTGGACCGGTCGAGGAATGGTTGTAGGTTATACACCATTAGAACTGCCCCTCAAGAATTGAATATGACAAAGGAATTGAAAAAATGCATCAGCCGCTTTGCTACTAGAATAACAAGTGAGGACTACTGCCCAGCCGCTGTTGCAAGCAGTGATTTTGTGGGCAAAATTGTTAATGCAGAGATGAATTCTAAAACATTCTATACCCGATTTGAGCCTGCACTTTATAGTGAAGAAAAGTATCACTTATTTGTCAAATATCAGGAAAAAGTGCATCAAGACTACAATAATTCGCCGAAGTCCTTTAAGCGATTCCTATGTGATACGCCCTTTGGCCCTGAAGCGGTGTTGGGGACACAAGAATCATGGGAGCAGTTAAACAATTGGCAGCGAATGAAGCCAGGCGAGAAACTGAAGCATATGGGTCCTGTGCACGAATGCTACTACTATGAGGGTAAATTAATTGCCATCACTGTGTCAGATATCTTGCCCAGTGGTATATCTTCGGTATATTTTATTTGGGATCCAGACTACAGTAAATGGTCCCTAGGGAAGCTCAGTGCTTTGCGCAAACTTGCCATCATTCAAAGGACAAATCTGCAATACTATTACCTGGGGTACTATATTGAGGACTGTCCGAAGATGAATTATAAAGCAAACTATGGTGCGGAAGTACTGGATGTGTGTCGGCAGTAAATATATACCCATTGAAACCCATTCAGGGATATGATTTCTAGA

TCCCTAGGGAAGCTCAGTGCTTTGCGCGATCTTGCCATCATTCAAAGGACAAATCTG **WT-ATE1**

TCCCTAGGGAAGCTCAGTGCTTTGCGCAAACTTGCCATCATTCAAAGGACAAATCTG **ATE1-D277K (R & C)**

CAGATTTGTCCTTTGAATGATGGCAAGTTTGCGCAAAGCACTGAGCTTCCCTAGGGA **ATE1-D277K (reverse Sanger sequencing read)**

**Supplementary Tables**

**Table S1**: List of yeast strains used in this study

| **Strain** | **Genotype** | **Source** |
| --- | --- | --- |
| BY4741 | *MAT a; his3*Δ*; leu2*Δ*0; met15*Δ*0; ura3*Δ*0* | EUROSCARF |
| *ate1*Δ | *BY4741; MAT a; his3*Δ*; leu2*Δ*0; met15*Δ*0; ura3*Δ*0; YGL017W::kanMX* | EUROSCARF |
| AH109 | *MATa, trp1-901, leu2- 3, 112, ura3-52, his3-200, gal4△, gal80△, LYS2:GAL1_UAS_-GAL1_TATA_-HIS3, GAL2_UAS_-GAL2_TATA_-ADE2, URA3:MELI_UAS_- MEL1_TATA_-lacZ* | Takara Bio |

**Table S2:** List of plasmids used in this study

| **Plasmid** | **Description** | **Source** |
| --- | --- | --- |
| pYES-ATE1-GFP | pYES2 *GAL1-promoter, CYC1-terminator, URA3, Amp^R^ with ATE1^WT^::GFP* (TEV cleavage site linker) | Gifted by Dr. F. Zhang, University of Miami, Miami, FL, USA |
| pGPD2-β-reporter | pGPD2 *GPD-promoter, CYC1-terminator, LEU2, Amp^R^,* with β-reporter (DD-β15-GFP) | Gifted by Dr. F. Zhang, University of Miami, Miami, FL, USA |
| pYES-ATE1-C20S-GFP | pYES-ATE1-GFP with *GAL1- ATE1^C20S^::GFP* (TEV cleavage site linker), *URA3, Amp^R^* | This study |
| pYES-ATE1-C23S-GFP | pYES-ATE1-GFP with *GAL1- ATE1^C23S^::GFP* (TEV cleavage site linker), *URA3, Amp^R^* | This study |
| pYES-ATE1-C20S-C23S-GFP | pYES-ATE1-GFP with *GAL1- ATE1^C20S,C23S^::GFP* (TEV cleavage site linker), *URA3, Amp^R^* | This study |
| pYES-ATE1-D277A-GFP | pYES-ATE1-GFP with *GAL1- ATE1^D277A^::GFP* (TEV cleavage site linker), *URA3, Amp^R^* | This study |
| pYES-ATE1-D277K-GFP | pYES-ATE1-GFP with *GAL1- ATE1^D277K^::GFP* (TEV cleavage site linker), *URA3, Amp^R^* | This study |
| pRS423-GAL- Trunc. Ate1-1-313aa | pRS423-GAL; *GAL1-promoter with GAL1-ATE1^1-313aa ,^ HIS3, Amp^R^* | This study |
| pYES- Trunc. Ate1-314-503aa | pYES-ATE1-GFP; *GAL1-promoter, with GAL1- ATE1^314-503aa^, URA3, Amp^R^* | This study |
| pYES-Trunc. Ate1-313aa-GFP | pYES-ATE1-GFP with *GAL1- ATE1^1-313aa^::GFP* (TEV cleavage site linker), *URA3, Amp^R^* | This study |
| pYES-Trunc. Ate1-364aa-GFP | pYES-ATE1-GFP with *GAL1- ATE1^1-364aa^::GFP* (TEV cleavage site linker), *URA3, Amp^R^* | This study |
| pYES- ATE1-314-503aa-GFP | pYES-ATE1-GFP with *GAL1- ATE^314-503aa^:: GFP* (TEV cleavage site linker), *URA3, Amp^R^* | This study |
| pRS423-ATE1-365-503aa-GFP | pRS423-GAL; *GAL1-promoter with GAL1-ATE1^365-503aa^::GFP, HIS3, Amp^R^* | This study |
| pYES-mouse-Ate1 | pYES2 *GAL1-promoter, CYC1-terminator, URA3, Amp^R^ with mouse Ate1* | This study |
| pGADT7-AD | *pGADT7-GAL4 AD, ADH1-promoter, SV40 NLS, T7-promoter, HA-tag, MCS, ADH1-terminator, LEU2, AmpR, pUC ori, 2µ ori.* | Takara Bio |
| pGBKT7-BD | *pGBKT7-GAL4 BD, ADH1-promoter, T7-promoter, c-Myc tag, MCS, T7 terminator, ADH1-terminator, TRP1, KanR, pUC ori, 2µ ori, f1 ori.* | Takara Bio |
| pGADT7-Ate1-1-313aa | *pGADT7-GAL4 AD; ADH1-promoter, with ATE1^1-313aa^ in frame with GAL4 AD,SV40 NLS, T7-promoter, HA-tag, , ADH1-terminator, LEU2, AmpR, pUC ori, 2µ ori.* | This study |
| pGBKT7-Ate1-314-503aa | *pGBKT7-GAL4 BD, ADH1-promoter, ATE1^314-503aa^ in frame with GAL4 BD, T7-promoter, c-Myc tag, T7 terminator, ADH1-terminator, TRP1, KanR, pUC ori, 2µ ori, f1 ori.* | This study |
| Stsp6A-pGADT7 | *pGADT7-GAL4 AD; ADH1-promoter, with Stsp6A in frame with GAL4 AD,SV40 NLS, T7-promoter, HA-tag, , ADH1-terminator, LEU2, AmpR, pUC ori, 2µ ori.* | Gifted by Dr. JP Maurya, Banaras Hindu University, Uttar Pradesh |
| StABL1-pGBKT7 | *pGBKT7-GAL4 BD, ADH1-promoter, StABL1 in frame with GAL4 BD, T7-promoter, c-Myc tag, T7 terminator, ADH1-terminator, TRP1, KanR, pUC ori, 2µ ori, f1 ori.* | Gifted by Dr. JP Maurya, Banaras Hindu University, Uttar Pradesh |

**Table S3:** List of primers used in this study

| **Oligos name** | **Sequences (5’-3’)** |
| --- | --- |
| Trunc.Ate1-EcoRI-F | GCAGAGATGAATTCTAAAACATTCTATACCCG |
| Trunc.Ate1-313aa-XhoI-R | ACTGTCCTCGAGATCCAGTACTTCCGCACCATAGTTTGC |
| Trunc. Ate1-364aa-Xho1-R | ACTGTCCTCGAGATCAGTGGGAAATCCCTCCCCTCTTCC |
| Trunc.ATE1-313aa-SpeI-F | TCGACTACTAGTATGTCCGATAGATTCGTTATTTGG |
| Trunc.ATE1-313aa-SmaI-R | AGCTCGCCCGGGTCAATCCAGTACTTCCGCACCATAGTTTG |
| Trunc.Ate1_314 to 503aa-SpeI-F | AGCTCGACTAGTATGGTGTGTCACAGTAAATATATC |
| Trunc.Ate1_314 to 503aa-SmaI-R | TCGACTCCCGGGTCACATTTGCTCACTATATAAAATG |
| ATE1-HindIII-F | GATATTAAGCTTGGTACCGCCATGTCCGATAGATTCG |
| F-Primer D277K | GGGAAGCTCAGTGCTTTGCGCAAACTTGCCATCATTCAAAGGAC |
| R-Primer D277K | GTCCTTTGAATGATGGCAAGTTTGCGCAAAGCACTGAGCTTCCC |
| Forward Primer D277A | GGGAAGCTCAGTGCTTTGCGCGCTCTTGCCATCATTCAAAGGAC |
| Reverse Primer D277A | GTCCTTTGAATGATGGCAAGAGCGCGCAAAGCACTGAGCTTCCC |
| ATE1-SacI-R | TGCATGGAGCTCCTTAGTAACTTTCGTCTCTTCTTC |
| ATE1-C20S-F | AATGAACCTGCCGCAAAGTCTGGGTATTGTCACGGTAATA |
| ATE1-C20S-R | TATTACCGTGACAATACCCAGACTTTGCGGCAGGTTCATT |
| ATE1-C23S-F | GCCGCAAAGTGTGGGTATTCTCACGGTAATAAGGGGGGC |
| ATE1-C23S-R | GCCCCCCTTATTACCGTGAGAATACCCACACTTTGCGGC |
| ATE1-C20S-C23S-F | CACAATGAACCTGCCGCAAAGTCTGGGTATTCTCACGGTAATAAGGGGGGCAAC |
| ATE1-C20S-C23S-R | GTTGCCCCCCTTATTACCGTGAGAATACCCAGACTTTGCGGCAGGTTCATTGTGC |
| ATE1-EcoRI-R | TGCATGGAATTCATCTCTGCATTAACAATTTTGCCC |
| Trunc. Ate1 314_503aa-HindIII-F | AGCTCGAAGCTTATGGTGTGTCACAGTAAATATATC |
| Trunc. Ate1 314_503aa-Xho1-R | TCGACTCTCGAGCATTTGCTCACTATATAAAATG |
| ATE1-365aa-EcoRI-F | AGCTCGGAATTCATGAATGTTGTCAAGTACAAGAATATTGC |
| Trunc. ATE1-1-313aa-SmaI-F | TCGACTCCCGGGATGTCCGATAGATTCGTTATTTGG |
| Trunc. ATE1-313aa-XhoI-R | AGCTCACTCGAGTCAATCCAGTACTTCCGCACCATAG |
| Trunc. Ate1- 314 to 503aa-EcoRI-F | AGCTCGGAATTCATGGTGTGTCACAGTAAATATATC |
| Trunc. Ate1  503aa-SalI-R | GACCTAGTCGACTCACATTTGCTCACTATATAAAATG |
| ATEGFP-SalI-R | TGCAGTGTCGACTCACTTGTACAGCTCGTCCATGCC |
| Ate1-seq-200Bp-R | ACATCCATATGTTCCACGAAAGAGC |
| mATE1.1-BamHI-F | TCGGCTGGATCCATGGCTTCTTGGAGCGCGCCTTC |
| mATE1.1-XhoI-R | CGCTGCCTCGAGTCAGTGTCTGAACAGCAGCATCC |

**Table S4:** Stability and activity of Ate1 mutants

| **Mutant** | **Protein stability** | **Enzymatic activity based on RDD signal** |
| --- | --- | --- |
| Ate1-C20S | stable | inactive |
| Ate1-C23S | stable | active |
| Ate1-C20,23S | stable | inactive |
| Ate1- D277A | stable | inactive |
| Ate1- D277K | unstable | inactive |

**Table S5:** Stability and activity of Ate1 truncates

| **Truncation** | **Protein stability** | **Enzymatic activity based on RDD signal** |
| --- | --- | --- |
| Ate1 – 1-313 aa | unstable | inactive |
| Ate1 – 314-503 aa | stable | inactive |
| Ate1 – 1-364 aa | stable | inactive |
| Ate1 – 365-503 aa | unstable | inactive |

**Supplementary figures:**

**Figure S1: The image shows the two Pfam domains identified in the Ate1, namely ATE_N and ATE_C, respectively.**

**Figure S2: Multiple sequence alignment performed using Clustal Omega shows that the C-terminal half of Ate1 is less conserved among eukaryotes.**

**Figure S3: Schematic representation of the *in vitro* arginylation reaction.** The *ate1*∆ cells were transformed with arginylation-reporter and Ate1 enzyme (wild-type, mutated or truncated), and the cell lysate was prepared. The arginylation reporter is a well-known substrate of Ate1, consisting of 15 amino acids of β-actin sequence, starting with aspartic acid and linearly fused with ubiquitin at the N-terminus. The reporter is also tagged with GFP at the C-terminus to further check its expression. To perform an *in vitro* arginylation reaction, equal volumes of lysates from *ate1*Δ cells expressing Ate1 and *ate1*Δ cells expressing the arginylation substrate (DD-β15-GFP) were mixed and underwent incubation at 37°C for 1 hour. Next, SDS sample buffer was added to stop the reaction and immunoblotting was performed. Further to assess arginylation and the steady state level of the reporter protein, the membrane was probed with anti-RDD and anti-GFP antibodies, respectively.

**Figure S4: Image showing the effect of charge reversal of active site residues of Ate1 on enzyme activity and Ate1-mediated cell death.** (A) Immunoblots showing arginylation and steady-state levels of the Ate1 substrate – DD-β15-GFP (arginylation reporter) in *ate1*Δ cells expressing the wild-type and Ate1-D277K mutants of Ate1. The stability of GFP-tagged Ate1 constructs was assessed using an anti-GFP antibody. The *ate1*Δ yeast cell transformed with the vector acts as a negative control for this *in vitro* arginylation assay. Pgk1, a cytosolic enzyme, was used as a loading control. (B) Spotting assay depicting the growth pattern of *ate1*Δ yeast cells transformed with vector, wild-type Ate1 and the Ate1 mutant (D277K) on SD-glucose (left panel, repressible condition) and SD-galactose (right panel, inducible condition) plates, respectively.

**Figure S5:** Image showing the presence of only one Caspase-1 (Casp1) cleavage site at Asp364 in *sc*Ate1.

**Figure S6: Co-expression of stable Ate1 truncation regions failed to reconstitute enzymatic activity *in vivo*.** Immunoblot showing the arginylation signal in the *ate1∆* yeast cell expressing the full-length Ate1 or its N and C-terminal halves with the indicated amino acid length. Pgk1, a cytosolic enzyme, act as a loading control under the given experimental condition.
